# Investigation into biomarkers to predict preterm birth (INSIGHT): a longitudinal UK pregnancy cohort for spontaneous preterm birth research

**DOI:** 10.3389/frph.2026.1926506

**Published:** 2026-09-30

**Authors:** Raphaella Jackson, Carlotta Valensin, Evonne Chin-Smith, Natalie Suff, Andrew H. Shennan, Natasha L. Hezelgrave, Rachel M. Tribe

**Affiliations:** Department of Women and Children's Health, King's College London, London, United Kingdom

**Keywords:** bioresource, cohort profile, longitudinal study, multi-omics, pregnancy, preterm (birth)

## Abstract

**Background:**

Spontaneous preterm birth (sPTB) remains a leading cause of perinatal morbidity and mortality worldwide. Progress in prediction and prevention has been limited by the biological heterogeneity of sPTB and a lack of longitudinal pregnancy cohorts integrating detailed clinical phenotyping with serial biological sampling. The INSIGHT study was established to enable mechanistic and biomarker-driven investigation of sPTB, with a focus on inflammatory pathways associated with cervical shortening.

**Methods:**

INSIGHT is a prospective, multicentre UK pregnancy cohort that was active between 2013 and 2024 (recruitment finished in 2023). Pregnant women were recruited between 10 ^+^ ^0^ and 28 ^+^ ^0^ weeks’ gestation and classified as high- or low-risk of preterm birth using predefined clinical criteria, including obstetric history, cervical length, prior cervical procedures, multiple pregnancy, and uterine anomalies. Serial clinical data were collected alongside longitudinal biological sampling, including maternal blood and cervicovaginal specimens, to support multiomic analyses. Pregnancy, delivery, and neonatal outcomes were ascertained through prospective data collection and routine clinical records.

**Results:**

A total of 2,272 women were enrolled and completed the study, spanning a broad sociodemographic background and risk spectrum, with 1,317 (58%) classified as high-risk of preterm birth. The cohort captured a high incidence of sPTB in singleton pregnancies (*n* = 184/2,184, (8.4%)), alongside detailed treatment, delivery, and neonatal outcome data. Extensive biological material was collected longitudinally, enabling transcriptomic, microbiome, metabolomic, proteomic, and immune profiling across pregnancy. A total of 34,863 specimens were collected over 4,999 participant sampling visits. Descriptive characteristics of participants, risk factor distributions, pregnancy outcomes, and sample collection are presented.

**Conclusion:**

INSIGHT provides a large, deeply phenotyped pregnancy cohort enriched for sPTB, integrating longitudinal clinical data with serial multiomic biological sampling. The study infrastructure supports discovery-driven and translational research into the mechanisms underlying sPTB and the development and independent validation of clinically scalable biomarker-based prediction strategies.

## 1 Introduction

Preterm birth, defined as birth before 37 weeks of gestation, is one of the most complex and pressing challenges in modern obstetric medicine. It is a leading cause of neonatal morbidity and mortality worldwide, with substantial long-term consequences for affected children and significant economic and health system burdens (1–4). Despite decades of research, the mechanisms of preterm birth remain poorly understood due to the complex and heterogeneous aetiologies involved (5). Current interventions and predictive tools remain limited in accuracy and scope, highlighting the need for further developments to effectively manage this global health concern (6, 7).

The INSIGHT cohort study was established to address this need by creating a deeply phenotyped cohort with comprehensive longitudinal data and biological sampling to investigate the mechanisms of sPTB in women at higher risk of early delivery due to obstetric and medical history. This risk group has higher rates of preterm birth, particularly early preterm birth, and mid-trimester loss (8–10) and results in some of the most severe consequences for affected mothers and children.

Mechanisms driving early sPTB are multifactorial but are most likely to be associated with a complex interaction of maternal and/or fetal inflammatory responses which culminate in the final common pathway of progressive cervical shortening and myometrial contractions (11). The principal aim of the study was to link innate immune function and host response with vaginal microbiome populations and gene polymorphisms. To achieve this, women at low- and high-risk of preterm birth were recruited to provide clinical data and a range of biological samples were collected from participants at various timepoints throughout their pregnancy including: cervicovaginal cells/fluid/mucus and blood.

INSIGHT was designed to address the need for prospective, longitudinal studies of women at increased risk of sPTB, with recruitment from early pregnancy. It was established to overcome important limitations of earlier studies, many of which focused on single timepoints or individual biological pathways in isolation. By recruiting a cohort enriched for women at increased risk of sPTB and combining serial biological sampling with detailed prospective phenotyping of pregnancy and neonatal outcomes, INSIGHT enables the interacting mechanisms underlying progressive cervical shortening and preterm labour to be investigated together rather than as separate processes. This integrated approach provides a unique opportunity to generate mechanistic insights in the population where they are most urgently needed.

In this paper, we describe the design, recruitment strategy, participant characteristics, biological sampling framework, and outcome structure of the INSIGHT pregnancy cohort. We present baseline descriptive data and an overview of pregnancy outcomes to illustrate the scope and scientific utility of the resource. Together, this work provides context for ongoing and future analyses aimed at improving mechanistic understanding and enabling clinically scalable prediction of sPTB.

## 2 Methods

### 2.1 Setting

The INSIGHT study is a prospective cohort study which aimed to determine the biological mechanisms underlying cervical shortening and sPTB, along with identifying novel biomarkers which could identify women at highest risk for preterm birth early in their pregnancy. The study was primarily conducted at Guy's and St Thomas’ NHS Foundation Hospital Trust (GSTT) in London with additional recruitment at St. Mary's Hospital (Manchester NHS Foundation Trust), St Mary's Hospital (University Hospitals Dorset NHS Foundation Trust), and West Middlesex University Hospital (Chelsea and Westminster Hospital NHS Foundation Trust). Recruitment took place between 2013 and 2023, with follow-up and sample collection continuing until 2024. This study is reported in accordance with the Strengthening the Reporting of Observational Studies in Epidemiology (STROBE) guidelines for cohort studies. The completed checklist is provided in the Supplementary Materials.

### 2.2 Recruitment and eligibility

INSIGHT recruited pregnant women from 10 **^+^** **^0^** to 28 ^+^ ^0^ weeks’ gestation based on last menstrual period, unless ultrasound dating was already available. For participants who subsequently had a dating ultrasound and did not miscarry before that scan, gestational age was updated using the ultrasound-estimated date of delivery. Unless otherwise stated, gestational ages reported throughout the manuscript are based on the final gestational age adjusted by ultrasound dating. Individuals were classified into two groups according to their risk of preterm birth. High-risk status was defined by the presence of one or more of the following criteria:

• Previous preterm birth or mid-trimester loss between 16 ^+^ ^0^ and 36 ^+^ ^6^ weeks’ gestation (mid-trimester loss between 14 ^+^ ^0^ and 15 ^+^ ^6^ weeks’ gestation is not included in this criterion).
• Previous preterm prelabour rupture of the fetal membranes (PPROM) at ≤ 36 ^+^ ^6^ weeks’ gestation
• Short cervical length (≤ 25 mm) on ultrasound between 18 ^+^ ^0^ and 24 ^+^ ^0^ weeks’ gestation
• Previous cervical procedure to treat abnormal smears (large loop excision, laser conisation, cold knife conisation, or radical diathermy)
• Multiple pregnancy
• Known Müllerian Abnormalities

Low-risk participants were those who met none of the above criteria. Women were excluded if there were known significant congenital, structural, or chromosomal fetal abnormalities. Potential high-risk participants were identified from referral letters to, or attendance at, preterm birth clinics, while potential low-risk participants were identified through attendance at antenatal clinics, early pregnancy units, or antenatal day units. In both cases, prospective participants were informed of the study and offered the opportunity to take part. Figure 1 shows the flow of participants from consent through to enrolment, together with total recruitment by site. A site-specific count of participant flow is available in Supplementary Table A.

**Figure 1:**
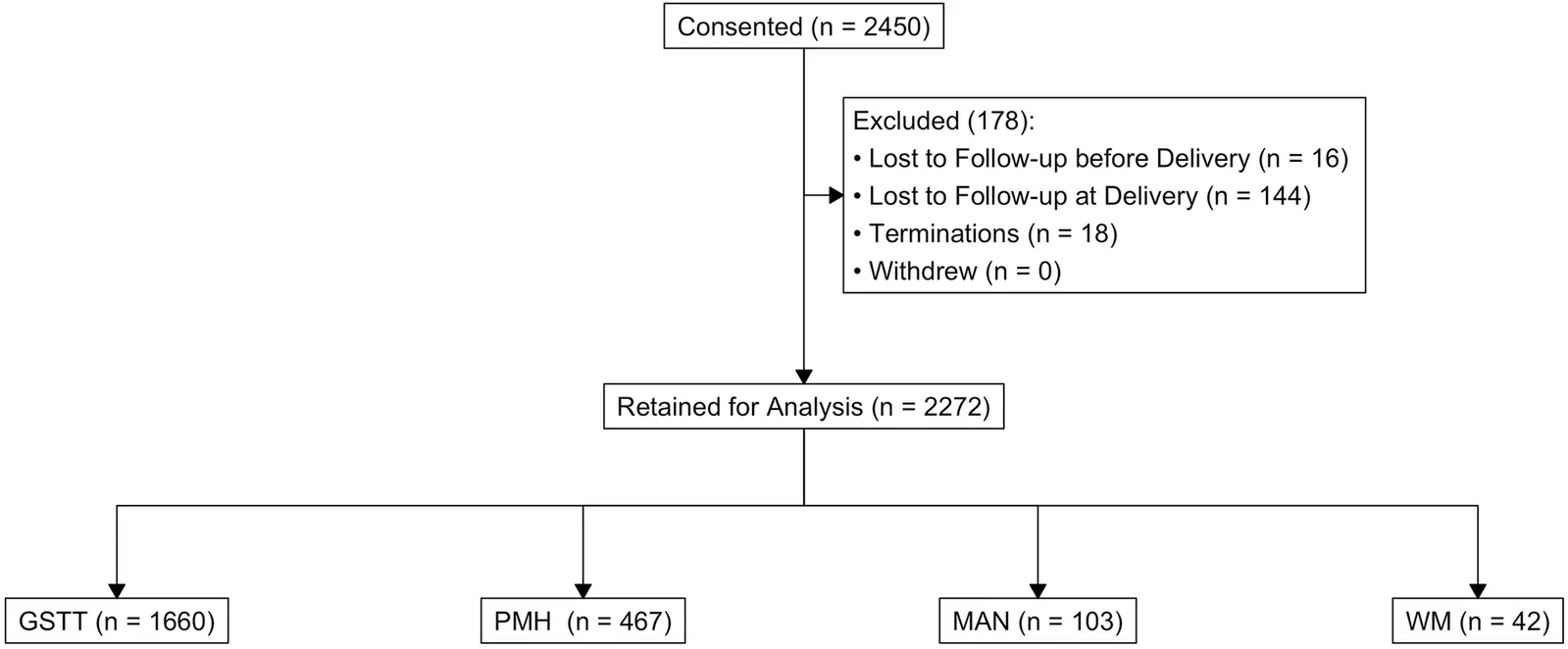
Participant flow diagram. Flow of participants from consent to inclusion in the analysis cohort, with retained participants stratified by recruitment site. “Lost to follow-up before delivery” refers to participants who consented but did not complete any research visits. “Lost to follow-up at delivery” refers to participants for whom basic delivery outcome data, including gestation at delivery and mode of delivery, could not be collected. GSTT denotes Guy’s and St Thomas’ NHS Foundation Trust, London; MAN denotes St Mary’s Hospital, Manchester University NHS Foundation Trust; PMH denotes St Mary’s Hospital, University Hospitals Dorset NHS Foundation Trust; WM denotes West Middlesex University Hospital, Chelsea and Westminster Hospital NHS Foundation Trust. A site-specific breakdown of consented, excluded, and retained participants is provided in Supplementary Table A.

### 2.3 Sample size and power calculations

The study aimed to recruit approximately 2,000 participants across high- and low-risk cohorts. Sample sizes for INSIGHT cohort recruitment were calculated using elafin as an exemplar candidate biomarker because sufficient preliminary data were available to support power calculations.

High-risk cohort (*n* ≈ 1,000): The sample size was based on validating cervical elafin (>200 ng/mL at 10 ^+^ ^0^–22 ^+^ ^6^ weeks’ gestation) as a predictor of short cervix (≤ 25 mm) and spontaneous preterm birth (sPTB). Approximately 400 participants were expected to develop a short cervix and 300 to deliver before 34 ^+^ ^0^ weeks. This sample provides 93% power to distinguish 50% from 40% sensitivity for sPTB and 99.98% power to distinguish 70% from 60% specificity. Recruitment estimates assumed 200 participants annually over five years.

Low-risk cohort (*n* ≈ 1,000): Recruitment aimed to match or exceed the high-risk cohort because of the lower prevalence of preterm birth (approximately 8%) in this population.

### 2.4 Data collection and clinical variables

Baseline sociodemographic, clinical, and behavioural data were collected at enrolment to characterise participants, support risk stratification, and enable downstream analyses. Sociodemographic variables included age at registration, self-reported ethnic background, and residential Lower-Layer Super Output Area (LSOA), allowing linkage to area-level indices of deprivation using the UK Index of Multiple Deprivation (IMD). Anthropometric measurements (height and weight) were recorded at or near enrolment, and body mass index (BMI) was derived. Ethnicity was self-reported and coded using categories informed by Office for National Statistics (ONS) classifications defined at study outset. For reporting, legacy category labels were updated to contemporary terminology: “AfroCaribbean” is reported as “Black Caribbean” and “Far East Asian” as “East Asian”; the underlying participant classifications are unchanged.

Clinical data relating to obstetric history and the index pregnancy were collected prospectively from participant interview and routinely recorded clinical information. These included gravidity and parity, singleton or multiple pregnancy status, gestational age at recruitment, and the presence of predefined risk factors for preterm birth used to classify participants as high- or low-risk at enrolment. Relevant medical history, including chronic hypertension, asthma, diabetes (type 1 and type 2), autoimmune disease, chronic renal disease, and chronic viral infection, was recorded due to their established associations with adverse pregnancy outcomes.

Medication use at enrolment was documented as a marker of underlying disease status and treatment burden, including antihypertensive agents, steroids, immunosuppressive therapies, and antibiotics. Behavioural and social exposures known to be associated with preterm birth risk were also collected, including smoking status, recreational drug use, and reported history of domestic violence. Infection-related history, such as recurrent urinary tract infection and previous bacterial vaginosis, was recorded given its relevance to inflammatory pathways implicated in sPTB.

Follow-up clinical data throughout pregnancy were collected through scheduled study visits, specialist antenatal appointments, and review of routinely collected maternity records. These data were used to capture pregnancy management, interventions, and outcomes, including cervical length measurements where available, antenatal admissions, and obstetric treatments administered during pregnancy.

A full list of recorded clinical variables is available in Supplementary Table B.

Demographic data along with medical and obstetric history were recorded in the study database. This database is an internet-based data management system designed and built by MedSciNet (now Omda), who are certified to provide these platforms for clinical trials, biobanks, and similar applications.

### 2.5 Biological sample collection and processing

Longitudinal biological sampling was conducted to support integrated multiomic analyses across pregnancy. Core samples were collected across four gestational-age windows — Recruitment to 13 ^+^ ^6^, 14 ^+^ ^0^–15 ^+^ ^6^, 16 ^+^ ^0^–19 ^+^ ^6^, and 20 ^+^ ^0^–24 ^+^ ^6^ weeks, with participants sampled at enrolment and again around the time of the mid-pregnancy anomaly scan, and at additional routine antenatal or specialist preterm birth clinic visits where feasible. The number and type of samples collected varied by participant and gestational age, reflecting clinical pathways and participant consent. A simplified summary of sample source and type is provided below in Table 1.

**Table 1:** Specimen Source Summary.

Sample Source	Derived Sample	Manuscript Sample Name
Blood in serum separator tube (SST)	Serum	Serum
Blood in tube containing ethylenediaminetetraacetic acid (EDTA)	Plasma	Plasma
Blood in tube containing ethylenediaminetetraacetic acid (EDTA)	Buffy coat	Buffy coat
Microbiology Swab 1	Swab smear on glass slide	Microbiology slide
Microbiology Swab 1	Swab suspension in liquid Amies	Swab stored in liquid Amies
Microbiology Swab 2	Swab suspension in TE buffer	Swab stored in TE buffer
High vaginal swab	Cervicovaginal fluid	High vaginal fluid
High vaginal swab	Cell Pellet	High vaginal cell pellet
Low vaginal swab	Cervicovaginal fluid	Low vaginal fluid
Low vaginal swab	Cell Pellet	Low vaginal cell pellet
Cytobrush	Cytobrush fluid	Cytobrush fluid
Cytobrush	Cell Pellet	Cytobrush cell pellet

Biological specimen types collected in the INSIGHT study, showing the sample source, the derived sample generated from that source, and the simplified sample name used throughout the manuscript and associated outputs. SST denotes serum separator tube; EDTA denotes ethylenediaminetetraacetic acid; TE denotes Tris-EDTA buffer. Microbiology swabs labelled “1” and “2” indicate two separate swabs collected using the same sampling procedure; the numbering distinguishes their downstream processing or storage purpose rather than a difference in collection method.

The core 10 ^+^ ^0^–24 ^+^ ^6^-week sampling window reflected clinical feasibility, from initial booking to completion of preterm birth surveillance, and the study focus on early biomarkers predictive of spontaneous preterm birth. Participants recruited after 24 ^+^ ^6^ weeks (up to the 28 ^+^ ^0^-week limit) entered clinical follow-up and contributed outcome data, but fell outside this core sampling schedule and are therefore not represented in the biological sampling shown in Figures 4, 5.

Samples were recorded in the MedSciNet study database and subsequently logged and tracked using FreezerPro® sample inventory management software (Azenta Life Sciences). Any thawing and refreezing events are recorded, allowing the suitability of individual samples to be assessed for each project according to the requirements of the intended analysis. Centralised sample-specific quality-control assays were not performed as part of biobanking. Instead, individual projects conduct the quality assessments appropriate to the specimen type and intended analysis following sample retrieval.

All samples except the cell pellet from the high vaginal swab and both specimens from the low vaginal swab were collected from the beginning of the study. Details on when the collection of additional swab specimens commenced is detailed below in the vaginal swab section.

#### 2.5.1 Vaginal swabs

Cervicovaginal fluid (CVF) was collected using low and high vaginal Dacron swabs. The low vaginal swab was positioned 2–3 cm from the introitus, while the high vaginal swab was placed in the posterior fornix during speculum examination. At each location, the swab was rotated through 360° and held in place for approximately 10 s to achieve saturation. Each swab was then placed in a separate tube containing 750 μL of phosphate-buffered saline supplemented with protease inhibitors (Complete; Roche Diagnostics GmbH, Germany) and transported immediately to the laboratory on ice.

In the laboratory, each swab was removed from its collection tube, transferred to a clean tube, vortexed for 10 s, and centrifuged at 2,600 × g for 10 min at 4 °C. The recovered fluid was combined with the fluid remaining in the corresponding collection tube, mixed, and centrifuged for a further 10 min to remove cellular debris. The resulting cell-free supernatants were divided into approximately 110-*μ*L aliquots and stored at −80 °C. This methodology is also reported in Hezelgrave *et al.* (12).

Time at collection and time at freezing were recorded for each sample. The protocol recommended processing samples within one hour of collection.

At the beginning of the study only the high vaginal swab was taken (not the low vaginal swab) and only the fluid was stored from the swab, the pellet was discarded. From February 2015 the cell pellet was retained and stored as described. The low vaginal swab was taken from May 2016, with both fluid and pellet being retained and stored as described.

#### 2.5.2 Microbiology swabs

Microbiology swabs were collected from the posterior fornix. Two swabs were collected at the same time. One was smeared onto a glass microscope slide and then placed in 1 mL of liquid Amies medium; the other was placed in 2 mL of TE buffer. Swabs in both tubes (liquid Amies and TE buffer) were rotated against the side of the tube for 30 s, after which the swabs were discarded. The tubes were then put on ice for transport to the laboratory and then stored at −80 °C. Glass microscope slides were air dried and then stored at room temperature.

Time at collection and time at freezing were recorded for each sample. The protocol recommended to process all samples within one hour of collection.

#### 2.5.3 Cytobrush samples

The cytobrush sample was taken by inserting the cytobrush in the cervix and rotating it 360° for 5 s. Each cytobrush was placed in a sterile 15 mL conical tube containing 750 μL of phosphate-buffered saline supplemented with protease inhibitors and transported to the laboratory on ice. Samples were processed within 1 h of collection.

In the laboratory, the cytobrush was gently agitated in the buffer and then removed from the tube. The remaining suspension was centrifuged at approximately 2,500 × g (approximately 4,000 rpm) for 10 min. The resulting cytobrush fluid (CBF) supernatant was divided into 150 μL aliquots, yielding approximately five aliquots per swab. The tube containing the cell pellet (CBP) was stored alongside the CBF aliquots at −80 °C.

Time at collection and time at freezing were recorded for each sample. The protocol recommended processing all samples within one hour of collection.

#### 2.5.4 Blood samples

A total of 15 mL of blood was collected via venepuncture into one tube containing ethylenediaminetetraacetic acid (EDTA) and one serum separator tube (SST). Following collection, the EDTA and SST-gel tubes were gently inverted several times to ensure thorough mixing without vigorous agitation.

In the laboratory, the SST-gel tube was allowed to stand at room temperature for 30 min before centrifugation. Both tubes were centrifuged at 2,500 × g (approximately 4,000 rpm) for 10 min at 4 °C. Plasma from the EDTA tube was divided into four 750 μL aliquots, while serum from the SST-gel tube was divided into three 750 μL aliquots. The buffy coat was removed from the EDTA tube using a Pasteur pipette and transferred to a new tube. Each microtube was stored at −80 °C.

Time at collection and time at freezing were recorded for each sample. The protocol recommended processing all samples within one hour of collection.

### 2.6 Outcomes and definitions

Pregnancy, delivery, and neonatal outcomes were ascertained prospectively using a combination of study data collection and routinely recorded clinical information. Preterm birth was defined as delivery before 37 ^+^ ^0^ weeks’ gestation and categorised according to clinical pathway and gestational age at delivery. Outcomes were classified as iatrogenic preterm birth or sPTB.

Additional pregnancy outcomes recorded included live birth, mid-trimester pregnancy loss, and stillbirth. Gestational age at delivery was recorded in completed weeks, alongside mode of onset of labour and mode of delivery. Preventive and acute obstetric interventions were documented, including use of progesterone, cervical cerclage, cervical pessary, tocolysis, antenatal corticosteroids, magnesium sulphate for fetal neuroprotection, antibiotics, amniocentesis, and *in utero* transfer.

Measures of healthcare utilisation were collected to characterise the clinical burden associated with threatened and established preterm birth. These included antenatal day unit attendances, antenatal and postnatal inpatient admissions, and total inpatient length of stay for the infant. Infection and inflammation related markers were also recorded, including diagnoses of bacterial vaginosis, abnormal maternal inflammatory markers (such as elevated C-reactive protein or white cell count), positive microbiological cultures, and placental histological evidence of chorioamnionitis where available.

Neonatal outcomes captured included birthweight, Apgar scores, major congenital abnormalities, evidence of neonatal infection, respiratory and neurological morbidity, requirement for oxygen or neonatal unit care at 28 days of life, inter-unit transfers, and neonatal death.

Together, these outcomes enable detailed characterisation of pregnancy course, delivery pathways, and short-term neonatal morbidity across gestational age groups. A full list of recorded outcome variables is available in Supplementary Table B.

### 2.7 Statistical analysis and visualisation

The data from the study was loaded and processed using R (version 4.6.1) (13). Dataset completeness was assessed using the package dlookr (version 0.6.5) (14). The participant flow diagram was generated using the package consort (version 1.2.3) (15). The map of participant LSOAs used the package sf (version 1.1–2) (16). Upset plots were generated using the package UpSetR (version 1.4.1) (17). All plots were made and the map was visualised using ggplot2 (version 4.0.3) (18). Tables were generated and mean/min/max/sd were calculated using the package gtsummary (version 2.5.1) (19). A full list of all software and dependencies used is available in the All Supplementary File. The All Supplementary File also gives the complete code and output of running the code used in compiling all figures and statistics in this paper.

Completeness of all variables in the dataset is reported in Supplementary Table B. Where variables are summarised or statistics are computed, the number of missing values is also reported. In all cases, “Not Reported” indicates that the information was unavailable in the participant's electronic healthcare record or that the participant declined to provide it; it does not reflect a decision by the study team to omit or not measure the information. “Not Measured” indicates that the relevant measurement was not performed.

### 2.8 Multisite study procedures and standardisation

All sites followed a single common study protocol for research activities. Data were captured using MedSciNet, a centrally controlled electronic data capture platform used by all sites, which supported consistent data entry and harmonisation across the study. To ensure consistency, a member of the central INSIGHT research team trained a lead midwife at each site and provided ongoing support, including regular check-ins.

## 3 Results

### 3.1 Recruitment and retention

Between 2013 and 2023, a total of 2,450 pregnant women were consented into the INSIGHT cohort across four NHS sites in the United Kingdom. Of these women, 2,272 remained in and completed the study. These 2,272 participants are described in the following sections. Participant flow from consent through enrolment and follow-up is summarised in Figure 1.

### 3.2 Baseline participant characteristics

Baseline sociodemographic and clinical characteristics of the cohort are summarised in Table 2. The mean age at recruitment was 32.8 years (SD 5.0; range 16.0–52.0 years). The cohort was ethnically diverse, with participants self-identifying as European (68%), African (13%), Black Caribbean (5.1%), and a range of Asian and Middle Eastern backgrounds, reflecting the population served by participating NHS sites. Area-level socioeconomic status, derived from residential LSOA, spanned the full Index of Multiple Deprivation (IMD) range (mean decile 4.8, SD 2.4).

**Table 2:** Sociodemographic, anthropometric, pregnancy, and recruitment characteristics of participants included in the INSIGHT study cohort.

Characteristic	*N* = 2,272^1^
Age at Registration	
Mean (SD)	32.8 (5.0)
Median (Q1, Q3)	33.0 (30.0, 36.0)
Min, Max	16.0, 52.0
Ethnic Background	
African	291 (13%)
Bangladeshi	7 (0.3%)
Black Caribbean	110 (5.1%)
East Asian	50 (2.3%)
European	1,474 (68%)
Indian	47 (2.2%)
Middle Eastern	10 (0.5%)
Other	116 (5.4%)
Pakistani	21 (1.0%)
South East Asian	38 (1.8%)
Not Reported	108 (4.8%)
BMI	
Mean (SD)	25.6 (5.4)
Median (Q1, Q3)	24.4 (21.8, 28.3)
Min, Max	12.9, 52.3
Not Reported	11 (0.5%)
Index of Multiple Deprivation (IMD) Decile (where 1 is most deprived 10% of LSOAs)	
Mean (SD)	4.8 (2.4)
Median (Q1, Q3)	4.0 (3.0, 6.0)
Min, Max	1.0, 10.0
Not Reported	102 (4.5%)
Gestation at Recruitment (Weeks)	
Mean (SD)	13.6 (2.1)
Median (Q1, Q3)	13.0 (12.3, 14.6)
Min, Max	8.0, 25.7
Primigravida	633 (28%)
Not Reported	6 (0.3%)
Pregnant with multiples	88 (3.9%)

1 *n* (%).

Percentages are calculated using the full participant cohort (*n* = 2,272) as the denominator unless otherwise indicated. Ethnicity was self-reported and coded using categories informed by Office for National Statistics (ONS) classifications defined at study outset. For reporting, legacy category labels were updated to contemporary terminology: “AfroCaribbean” is reported as “Black Caribbean” and “Far East Asian” as “East Asian”; the underlying participant classifications were unchanged. Gestation at Recruitment reflects final dating following ultrasound. Where early ultrasound redating revised the estimated gestation, the recorded value at recruitment may fall below the 10 ^+ 0^ week enrolment threshold, accounting for the minimum of 8.0 weeks shown.

The majority of pregnancies were singleton (*n* = 2,184, 96%), and 28% (*n* = 633) of participants were primigravid. Mean body mass index at recruitment was 25.6 kg/m^2^ (SD 5.4). The mean gestational age at enrolment was 13.6 weeks (SD 2.1). A supplementary breakdown comparing basic demographic characteristics of participants by site and by lost to follow-up versus those in the analysis cohort is provided in Supplementary Table C parts [A] and [B] respectively.

The geographic distribution of participants by residential lower-layer super output area (LSOA) is shown in Figure 2, illustrating the broad geographic reach of recruitment across participating sites.

**Figure 2:**
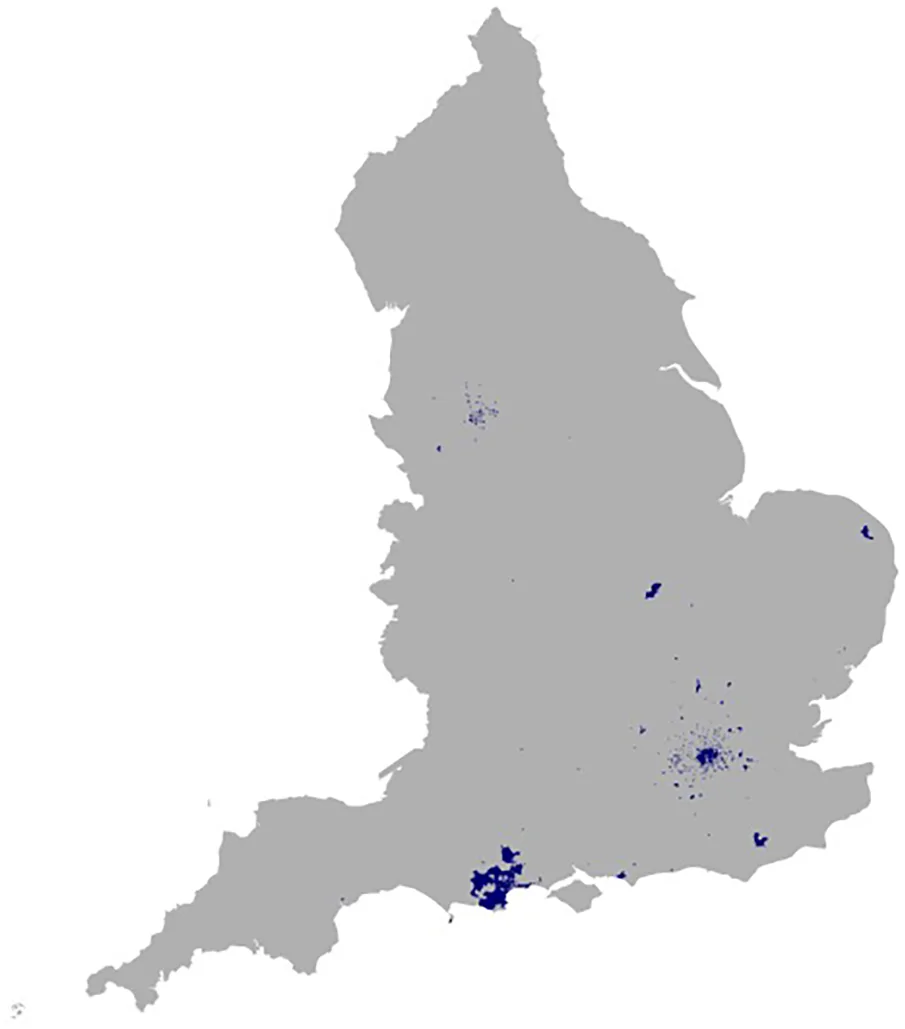
Geographic distribution of participants. Map showing lower layer super output areas (LSOAs) represented by participants in the INSIGHT study cohort. LSOAs containing at least one participant are shaded navy; all other English LSOAs are shaded grey. LSOA denotes lower layer super output area, a small-area geographic unit used for reporting population statistics in England.

### 3.3 Risk stratification and risk factor distribution

Based on the predefined clinical criteria, 1,317 women (58%) were classified as high-risk of preterm birth, while 955 women (42%) were classified as low-risk. The distribution and overlap of individual risk factors among high-risk participants are shown in Figure 3, with frequencies summarised in Supplementary Tables D, E. The reported frequencies are the final, updated risk status of participants, in cases where participants had a cervical measurement (≤ 25 mm) on ultrasound between 18 ^+^ ^0^ and 24 ^+^ ^0^ weeks’ gestation which occurred after their enrolment visit.

**Figure 3:**
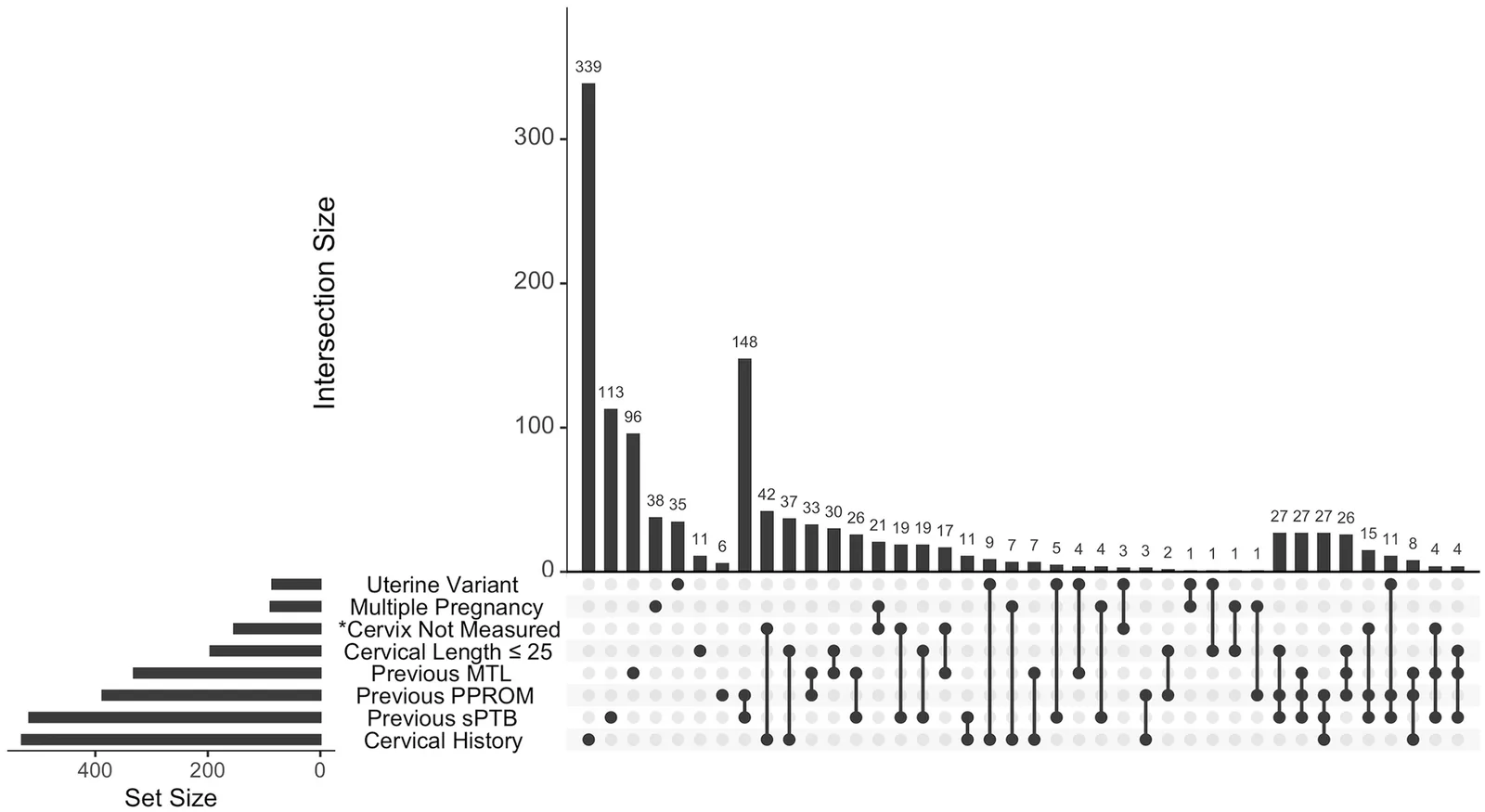
Distribution and overlap of preterm birth risk factors. UpSet plot showing the individual and overlapping risk factors used to classify participants as being at high-risk of preterm birth. Set size indicates the number of participants with each risk factor irrespective of other co-occurring risk factors; intersection size indicates the number of participants with each specific combination of risk factors. Solid black circles indicate risk factors present in each group, and connecting black lines indicate combinations involving more than one risk factor. SPTB denotes spontaneous preterm birth; MTL denotes mid-trimester loss between 16 ^+ 0^ and 23 ^+ 6^ weeks’ gestation (mid-trimester loss between 14 ^+ 0^ and 15 ^+ 6^ weeks’ gestation is not included in this criterion); PPROM denotes preterm prelabour rupture of membranes. Cervical history refers to previous cervical procedures. All risk factors are fully defined in the Recruitment and Eligibility section. The reported frequencies are the final, updated risk status of participants, in cases where participants had a cervical measurement ≤ 25 mm on ultrasound between 18 ^+ 0^ and 24 ^+ 0^ weeks’ gestation which occurred after their enrolment visit.

Risk factors were not mutually exclusive and frequently co-occurred. Cervical-related risk factors (short cervical length and/or previous cervical procedures) were common, often overlapping with a history of prior sPTB or mid-trimester pregnancy loss from 16 ^+^ ^0^ weeks’ gestation. Among high-risk participants with a recorded cervical length measurement between 18 ^+^ ^0^ and 24 ^+^ ^0^ weeks’ gestation (*n* = 1,164), 195 women (17%) had a cervical length of ≤25 mm.

### 3.4 Pregnancy and delivery outcomes

Pregnancy and delivery outcomes for singleton pregnancies in the cohort are summarised in Table 3. The same outcomes are summarised for twin pregnancies (the highest order of multiples within the INSIGHT cohort) in Supplementary Table F. Out of 2,272 women, 96% (2,184) in the INSIGHT cohort had singleton pregnancies. Most singleton pregnancies, 87%, resulted in a term birth. Preterm birth occurred in 11% (248) pregnancies, comprising more spontaneous preterm births (184) than iatrogenic preterm births (64). The majority of pregnancies resulted in live birth (98%), with stillbirth and loss before 24 weeks making up 0.6% and 1.7% of singleton pregnancies in total.

**Table 3:** Pregnancy and delivery outcomes among singleton pregnancies in the INSIGHT cohort.

[A] Pregnancy Outcomes—Singletons						
	Term, *N* = 1,899 (87%)^1^	iPTB, *N* = 64 (2.9%)^1^	sPTB, *N* = 184 (8.4%)^1^	Mid-trimester Loss, *N* = 36 (1.6%)^1^	Miscarriage, *N* = 1 (<0.1%)^1^	Overall, *N* = 2,184 (100%)^1^
High Risk	992/1,899 (52%)	43/64 (67%)	160/184 (87%)	34/36 (94%)	0/1 (0%)	1,229/2,184 (56%)
Pregnancy Outcome						
Live birth	1,897/1,899 (100%)	60/64 (94%)	176/184 (96%)	0/36 (0%)	0/1 (0%)	2,133/2,184 (98%)
Stillbirth	2/1,899 (0.1%)	4/64 (6.3%)	8/184 (4.3%)	0/36 (0%)	0/1 (0%)	14/2,184 (0.6%)
Loss Before 24 Weeks	0/1,899 (0%)	0/64 (0%)	0/184 (0%)	36/36 (100%)	1/1 (100%)	37/2,184 (1.7%)
Gestation at Delivery (Weeks)						
Mean (SD)	39.6 (1.2)	34.8 (2.2)	32.9 (4.2)	18.5 (2.1)	12.1 (NA)	38.5 (3.7)
Median (Q1, Q3)	39.6 (38.7, 40.6)	35.4 (34.2, 36.3)	34.6 (31.1, 36.2)	18.4 (17.0, 19.9)	12.1 (12.1, 12.1)	39.3 (38.1, 40.3)
Min, Max	37.0, 42.6	27.7, 36.9	20.6, 36.9	14.3, 23.6	12.1, 12.1	12.1, 42.6

[B] Neonatal Outcomes—Singletons				
	Term, *N* = 1,897 (89%)^1^	iPTB, *N* = 60 (2.8%)^1^	sPTB, *N* = 176 (8.3%)^1^	Overall, *N* = 2,133 (100%)^1^
Admission to SCBU or NICU	57/1,896 (3.0%)	32/60 (53%)	93/176 (53%)	182/2,132 (8.5%)
Not Reported	1/1,897 (<0.1%)	0/60 (0%)	0/176 (0%)	1/2,133 (<0.1%)
Number of inpatient nights				
Mean (SD)	1.9 (2.7)	12.6 (17.2)	22.5 (48.7)	3.7 (14.9)
Median (Q1, Q3)	1.0 (1.0, 2.0)	7.0 (4.0, 12.5)	7.0 (4.0, 15.0)	2.0 (1.0, 3.0)
Min, Max	0.0, 63.0	1.0, 94.0	1.0, 395.0	0.0, 395.0
Not Reported	69/1,897 (3.6%)	4/60 (6.7%)	23/176 (13%)	96/2,133 (4.5%)

1 *n*/*N* (%).

Outcomes are summarised for participants with singleton pregnancies. Table 3A presents pregnancy outcomes, and Table 3B presents neonatal outcomes. Values are presented as *n* (%) unless otherwise stated. Percentages in Table 3A are calculated using the number of singleton pregnancies within each outcome group as the denominator; for singleton pregnancies, this is equivalent to the number of participants. Percentages in Table 3B are calculated using the number of singleton live births within each outcome group as the denominator; miscarriages, stillbirths, and mid-trimester losses are excluded from these denominators because admission to SCBU or NICU and inpatient neonatal stay are not applicable to these outcomes, while applicable outcomes not reported are retained as “Not Reported”. Number of inpatient nights refers to the number of nights the infant remained admitted to hospital after birth. Term birth was defined as delivery at ≥ 37 ^+^ ^0^ weeks’ gestation. Spontaneous preterm birth (sPTB) was defined as delivery before 37 ^+^ ^0^ weeks’ gestation following spontaneous onset of labour or preterm prelabour rupture of membranes. Iatrogenic preterm birth (iPTB) was defined as delivery before 37 ^+^ ^0^ weeks’ gestation following medical intervention, including induction of labour or prelabour caesarean birth for maternal or fetal indications. Mid-trimester loss was defined as pregnancy loss between 14 ^+^ ^0^ and 23 ^+^ ^6^ weeks’ gestation. Stillbirth was defined as birth without signs of life at or beyond 24 ^+^ ^0^ weeks’ gestation. Miscarriage was defined as pregnancy loss before 14 ^+^ ^0^ weeks’ gestation with known outcome details; missed miscarriages are not included in this category. SCBU denotes Special Care Baby Unit; NICU denotes Neonatal Intensive Care Unit.

Gestational age at delivery differed across outcome groups. Mean gestational age was 39.6 weeks among term births, compared with 34.8 weeks for iatrogenic preterm births and 32.9 weeks for spontaneous preterm births. Adverse pregnancy outcomes were also associated with greater healthcare utilisation. Mean inpatient stay for the infant increased from 1.9 nights among term births to 12.6 nights for iatrogenic preterm births and 22.5 nights for spontaneous preterm births. Similarly, neonatal admission to a special care baby unit or neonatal intensive care unit occurred in only 3.0% of term births but in approximately half of both iatrogenic (53%) and spontaneous (53%) preterm births.

### 3.5 Sample collection

Longitudinal biological samples were collected from participants across pregnancy, including maternal blood and cervicovaginal specimens. The distribution of collected sample types shaded by window of gestational age at collection and stratified by primary pregnancy outcome, is shown in Figure 4. Sample availability varied by gestational age at delivery and engagement with follow-up visits, reflecting clinical pathways and attrition. A full table of counts of each sample type collected by gestation at collection is available in Supplementary Table G with a summary of average aliquots collected for each sample type in Supplementary Table H.

**Figure 4:**
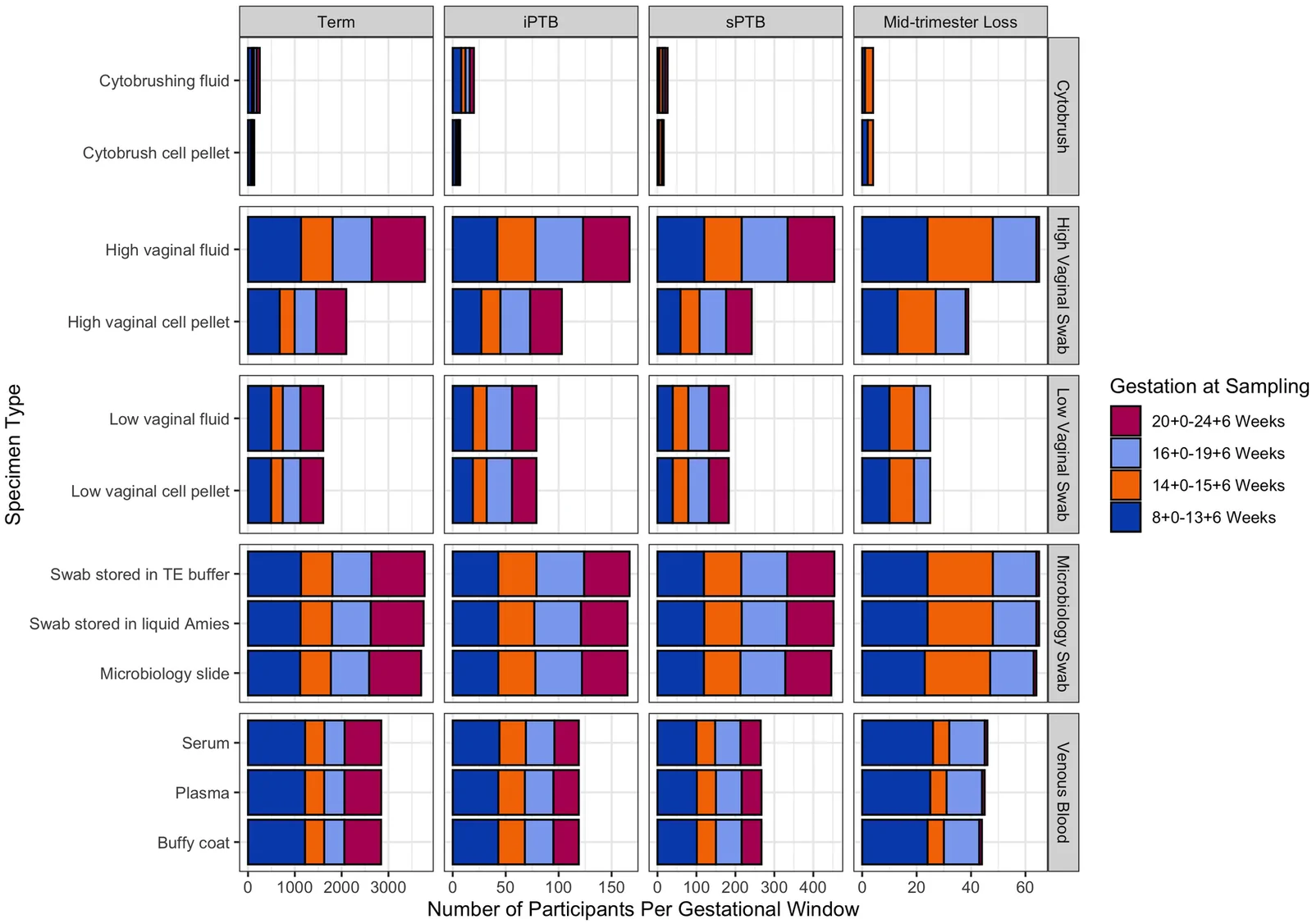
Biological specimen collection by pregnancy outcome. Distribution of participants contributing each specimen type, stratified by primary pregnancy outcome as defined in the Pregnancy and Delivery Outcomes section. Within each outcome stratum, each coloured segment represents the number of distinct participants who contributed at least one specimen of the specified type during the relevant gestational sampling window. A participant who contributed multiple specimens of the same type within a given window is counted only once in that segment. However, participants may be counted in multiple segments if they contributed specimens during more than one gestational window. Consequently, the total length of a stacked bar represents the sum of the window-specific participant counts and does not necessarily equal the number of unique participants who contributed that specimen type. Facet scales are allowed to vary for readability. One participant who experienced miscarriage and contributed three specimens at their first and only visit was omitted from the plot for readability; complete sample collection counts, including data from this participant, are provided in Supplementary Table G.

Cervicovaginal samples and blood specimens were successfully collected from the majority of participants at enrolment, with repeat sampling undertaken where feasible. The number of participants with visits in each gestational window (where they gave samples) is visualised in Figure 5, with the visualised data being given in a table format in Supplementary Table I. A summary of the number and outcomes of participants who did not end up giving any samples is available in Supplementary Table J.

**Figure 5:**
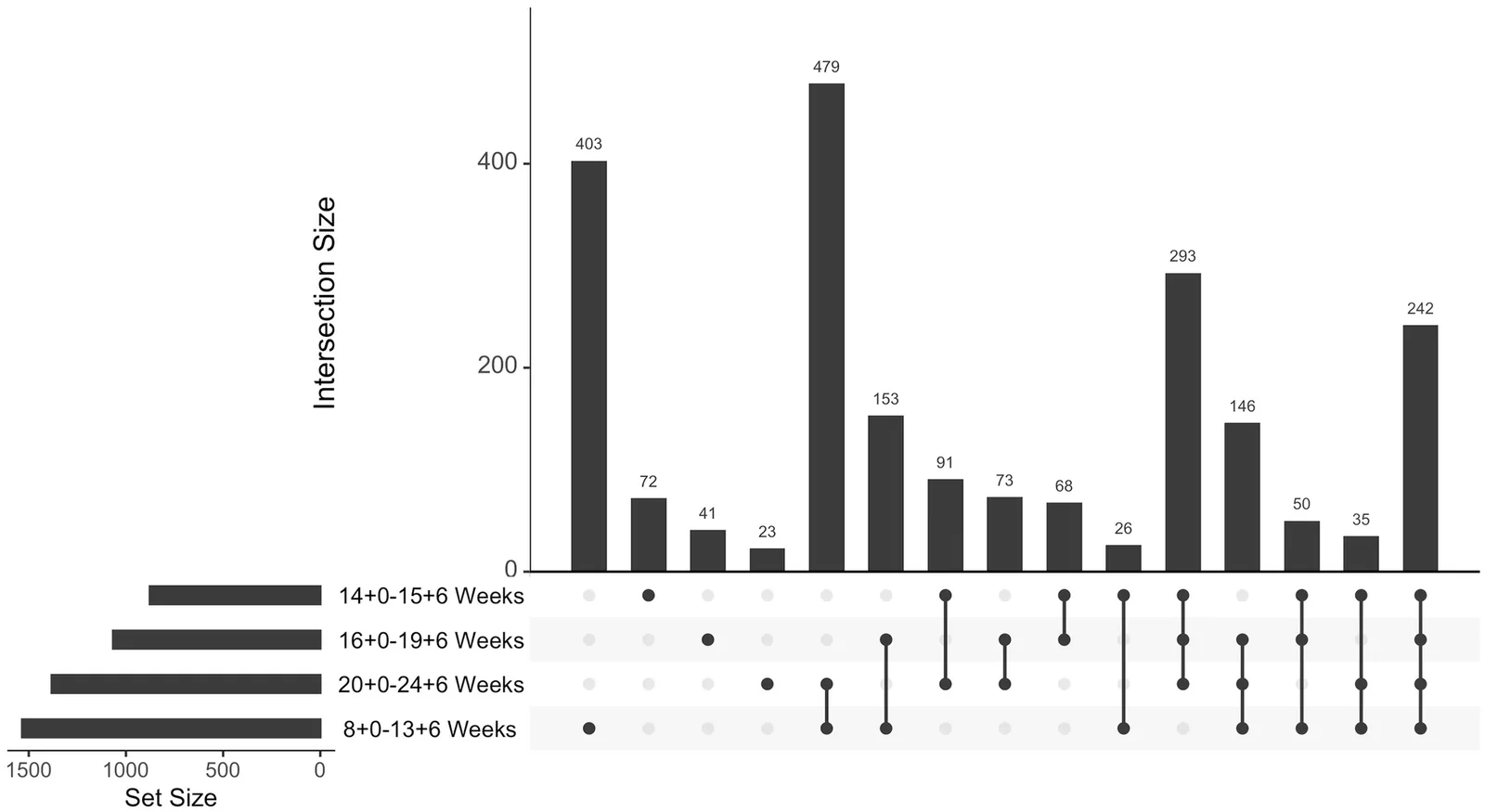
Participant sampling visit patterns. UpSet plot showing patterns of biological specimen contribution across gestational sampling windows in the INSIGHT cohort. Each set represents a gestational window in which participants contributed at least one biological specimen. Set size indicates the total number of participants who contributed a specimen within each window, irrespective of attendance at other windows. Intersection size indicates the number of participants who contributed specimens in each specific combination of gestational windows. Solid black circles indicate included sampling windows, and connecting black lines indicate participants who contributed specimens across more than one window.

Together, these samples support multiomic analyses spanning transcriptomic, microbiome, metabolomic, proteomic, and immune profiling and provide a richly characterised biological resource linked to detailed clinical outcome data.

## 4 Discussion

### 4.1 Strengths

The INSIGHT pregnancy cohort was established to address longstanding methodological and biological constraints in the study of sPTB, particularly at early gestational ages when morbidity and mortality are greatest. By integrating longitudinal recruitment with deep clinical phenotyping and serial biological sampling, INSIGHT provides a comprehensive platform for investigating the heterogeneous inflammatory, microbial, and host-derived pathways that contribute to sPTB. The descriptive results presented here illustrate the scale, outcome richness, and translational relevance of the cohort, while previously published INSIGHT outputs demonstrate the scientific value of this design in practice.

A central strength of INSIGHT is its size, duration, and prospective longitudinal structure. Recruitment over a ten-year period enabled enrolment of many participants across both high and low-risk groups, while repeated sampling across gestation allowed investigation of biological changes that precede, rather than simply accompany, clinical presentation. Importantly, INSIGHT was intentionally enriched for women at increased risk of early sPTB and mid-trimester pregnancy loss, conditions that account for a disproportionate share of adverse neonatal outcomes. This enrichment has ensured sufficient capture of relatively rare but clinically severe outcomes and has been instrumental in enabling meaningful mechanistic analyses focused on early disease processes.

The scientific outputs generated from INSIGHT to date provide concrete evidence that this design has delivered on its original aims. INSIGHT-based analyses have shown that blood-based transcriptomic profiling enables early identification of pregnancy complications, with maternal plasma cell-free RNA signatures detectable months before the clinical onset of sPTB (20). Related work demonstrated that these transcriptomic signals reflect placental, maternal, and fetal biology across gestation and support the broader potential of blood-based molecular tests for early prediction of adverse pregnancy outcomes (21, 22).

Complementary mechanistic findings from INSIGHT emphasise the importance of local host–microbe interactions at the cervicovaginal interface. Integrated early-pregnancy analyses of cervicovaginal microbiota, metabolites, and host immune features demonstrated improved prediction of sPTB and revealed coordinated host–microbe interactions linked to inflammatory pathways (23). Further work characterising cervicovaginal innate immune peptides, local immune cell composition, and the maternal inflammatory proteome showed that dysregulated immune responses across pregnancy are strongly associated with sPTB risk, supporting the presence of distinct biological pathways converging on a shared clinical outcome (12, 24–26).

These mechanistic findings have been further strengthened by work linking microbial activity to clinically relevant pregnancy phenotypes, strengthening the translational relevance of the cohort. Metabolomic analyses of bacterial vaginosis-associated communities identified metabolic strategies and microbial interactions capable of generating metabolites previously associated with preterm birth risk, providing mechanistic context for observed microbiome–outcome associations (27). In parallel, clinically focused analyses evaluating established predictors such as cervical length and quantitative fetal fibronectin in high-risk women, including those with cervical cerclage, demonstrated continued utility of these measures for real-world risk stratification and management, particularly in identifying women at risk of treatment failure (28).

### 4.2 Limitations

These strengths should be interpreted alongside several limitations. INSIGHT was deliberately designed to recruit pregnant women at increased risk of early sPTB and mid-trimester loss. Spontaneous preterm birth is a rare outcome in pregnancy, so this was necessary to recruit sufficient participants that would go on to experience sPTB. This recruitment strategy resulted in high-risk participants comprising 58% of the cohort. As a result this cohort is not representative of the general obstetric population, and observed outcome frequencies and biomarker associations should not be read as population-level estimates.

Recruitment was also concentrated in London and drew substantially on specialist referral clinics, with the majority of participants enrolled at a single site (1,660 of 2,272 recruits at GSTT), introducing potential selection and site-level effects and limiting generalisability to other settings, particularly outside the UK and the NHS. The cohort was predominantly of European ethnicity (68%), which further constrains the applicability of findings to more ethnically diverse populations in whom sPTB risk and its underlying biological mechanisms may differ.

An additional limitation of the study is the loss of some participants to follow-up. However, only a relatively small number of participants did not complete the study (7%, *n* = 160). The majority of those were lost to follow-up at delivery (6%, *n* = 144), with the remainder (*n* = 16) lost before delivery. An additional 18 pregnancies that ended in termination were excluded because they did not provide information relevant to the mechanisms of labour examined in this study. The main issue for consideration is that participants with unavailable delivery outcomes may not represent a random subset of the cohort. Those who went into labour early and unexpectedly may have been more likely to be lost to follow-up for reasons such as delivering in a different location than planned. As only limited data are available for these participants, it is not possible to assess if their outcomes differed systematically from the cohort as a whole and therefore a degree of bias cannot be ruled out.

Several further features of the cohort should be kept in mind in future investigations. As noted above, high-vaginal cell pellets were retained only from February 2015, and low-vaginal specimens from May 2016. Availability of these sample types is therefore partly determined by recruitment year, and therefore analyses using these sample types are necessarily confined to a later subset of the cohort. The presence of multiple pregnancies adds analytic complexity, as twin and higher-order pregnancies carry distinct preterm birth risks and violate the assumption of independent observations, and will require appropriate handling in any analysis. Some outcomes were derived from routine clinical records rather than research-specific data collection, which may introduce variability in completeness and coding; because recruitment was distributed unevenly across sites, this also raises the possibility of site-related differences in outcome completeness and ascertainment. Furthermore, although we have distinguished sPTB from iatrogenic delivery, stillbirth and pregnancy loss in the present analyses, future work should continue to phenotype these outcomes carefully, as grouping distinct delivery and loss mechanisms could obscure or bias biomarker associations.

Finally, screened and approached recruitment information was not retained for this study. However, we note that the self-reported ethnicity in our study reflects the local populations of Lambeth and Southwark which was our largest catchment area for the study and exhibits greater heterogeneity than the UK population as a whole (29).

## 5 Conclusions

Taken together, INSIGHT constitutes a large, deeply phenotyped UK pregnancy cohort enriched for sPTB, with a longitudinal clinical and biological data structure designed to support integrated mechanistic and methodological research. The descriptive results presented here, alongside previously published outputs derived from the cohort, demonstrate how early-pregnancy molecular, microbial, immune, and clinical data can be systematically captured and linked to pregnancy outcomes within a real-world healthcare setting. INSIGHT provides a robust framework for continued secondary analysis, including refinement of predictive approaches, characterisation of biologically heterogeneous pathways to sPTB, and evaluation of combined clinical and biomarker-based strategies. We emphasise, however, that the present manuscript is a descriptive account of the cohort and its resource; the predictive and biomarker-based findings referenced here derive from prior focused analyses, and clinically scalable prediction strategies will require dedicated validation in independent, representative populations before translation into practice.

## 6 Patient and public involvement statement

Patients attending the preterm birth surveillance clinic at GSTT have been involved in this research from its earliest stages through an established preterm birth Patient and Public Involvement and Engagement (PPIE) group, active since 2012. As the clinic is a referral centre, PPIE members are drawn from a wide geographic area and not solely from London. This group has helped shape research priorities, refine research questions, and identify outcomes that reflect patient concerns and preferences. Members have provided input into study design and participant materials, including consideration of participant burden and feasibility. They have also advised on recruitment approaches and will contribute to dissemination planning, including decisions about which findings to share with patient communities and in what formats.

## Data Availability

The data analysed in this study is subject to the following licenses/restrictions: INSIGHT data and samples constitute a controlled-access resource and are available through approved collaborations rather than through open or direct access. Requests to access these datasets should be directed to rachel.tribe@kcl.ac.uk.
