# Supplementary material for: Investigation into biomarkers to predict preterm birth (INSIGHT): a longitudinal UK pregnancy cohort for spontaneous preterm birth research
Source: Front Reprod Health. 2026 Sep 30;8:1926506. doi: 10.3389/frph.2026.1926506 (PMC13661963; doi:10.3389/frph.2026.1926506)
Supplement: Supplementary file 1 [file Table1.docx]

STROBE Statement—Checklist of items that should be included in reports of ***cohort studies***

|  | Item No | Recommendation |
| --- | --- | --- |
| **Title and abstract** | 1 | (*a*) Indicate the study’s design with a commonly used term in the title or the abstract |
|  |  | (*b*) Provide in the abstract an informative and balanced summary of what was done and what was found |
| Introduction | | |
| Background/rationale | 2 | Explain the scientific background and rationale for the investigation being reported |
| Objectives | 3 | State specific objectives, including any prespecified hypotheses |
| Methods | | |
| Study design | 4 | Present key elements of study design early in the paper |
| Setting | 5 | Describe the setting, locations, and relevant dates, including periods of recruitment, exposure, follow-up, and data collection |
| Participants | 6 | (*a*) Give the eligibility criteria, and the sources and methods of selection of participants. Describe methods of follow-up |
|  |  | (*b*) For matched studies, give matching criteria and number of exposed and unexposed |
| Variables | 7 | Clearly define all outcomes, exposures, predictors, potential confounders, and effect modifiers. Give diagnostic criteria, if applicable |
| Data sources/ measurement | 8* | For each variable of interest, give sources of data and details of methods of assessment (measurement). Describe comparability of assessment methods if there is more than one group |
| Bias | 9 | Describe any efforts to address potential sources of bias |
| Study size | 10 | Explain how the study size was arrived at |
| Quantitative variables | 11 | Explain how quantitative variables were handled in the analyses. If applicable, describe which groupings were chosen and why |
| Statistical methods | 12 | (*a*) Describe all statistical methods, including those used to control for confounding |
|  |  | (*b*) Describe any methods used to examine subgroups and interactions |
|  |  | (*c*) Explain how missing data were addressed |
|  |  | (*d*) If applicable, explain how loss to follow-up was addressed |
|  |  | (*e*) Describe any sensitivity analyses |
| Results | | |
| Participants | 13* | (a) Report numbers of individuals at each stage of study—eg numbers potentially eligible, examined for eligibility, confirmed eligible, included in the study, completing follow-up, and analysed |
|  |  | (b) Give reasons for non-participation at each stage |
|  |  | (c) Consider use of a flow diagram |
| Descriptive data | 14* | (a) Give characteristics of study participants (eg demographic, clinical, social) and information on exposures and potential confounders |
|  |  | (b) Indicate number of participants with missing data for each variable of interest |
|  |  | (c) Summarise follow-up time (eg, average and total amount) |
| Outcome data | 15* | Report numbers of outcome events or summary measures over time |
| Main results | 16 | (*a*) Give unadjusted estimates and, if applicable, confounder-adjusted estimates and their precision (eg, 95% confidence interval). Make clear which confounders were adjusted for and why they were included |
|  |  | (*b*) Report category boundaries when continuous variables were categorized |
|  |  | (*c*) If relevant, consider translating estimates of relative risk into absolute risk for a meaningful time period |
| Other analyses | 17 | Report other analyses done—eg analyses of subgroups and interactions, and sensitivity analyses |
| Discussion | | |
| Key results | 18 | Summarise key results with reference to study objectives |
| Limitations | 19 | Discuss limitations of the study, taking into account sources of potential bias or imprecision. Discuss both direction and magnitude of any potential bias |
| Interpretation | 20 | Give a cautious overall interpretation of results considering objectives, limitations, multiplicity of analyses, results from similar studies, and other relevant evidence |
| Generalisability | 21 | Discuss the generalisability (external validity) of the study results |
| Other information | | |
| Funding | 22 | Give the source of funding and the role of the funders for the present study and, if applicable, for the original study on which the present article is based |

*Give information separately for exposed and unexposed groups.

**Note:** An Explanation and Elaboration article discusses each checklist item and gives methodological background and published examples of transparent reporting. The STROBE checklist is best used in conjunction with this article (freely available on the Web sites of PLoS Medicine at http://www.plosmedicine.org/, Annals of Internal Medicine at http://www.annals.org/, and Epidemiology at http://www.epidem.com/). Information on the STROBE Initiative is available at http://www.strobe-statement.org.

| **Item** | **Recommendation** | **Addressed in manuscript** |
| --- | --- | --- |
| **1a** | Study design in title/abstract | Title states "longitudinal UK pregnancy cohort"; abstract describes INSIGHT as a "prospective, multicentre UK pregnancy cohort." |
| **1b** | Informative abstract | Abstract includes background, methods, results, and conclusions. |
| **2** | Background/rationale | Introduction paragraphs 1-4 explain burden of sPTB, knowledge gaps, and rationale for establishing INSIGHT. |
| **3** | Objectives | Introduction states aims to investigate biological mechanisms underlying sPTB, identify biomarkers, and establish a deeply phenotyped cohort. |
| **4** | Study design | Methods, Setting section describes INSIGHT as a prospective cohort study with longitudinal clinical and biological sampling. |
| **5** | Setting | Methods, Setting section describes sites, locations, recruitment period (2013-2023), follow-up to 2024, and data collection procedures. |
| **6a** | Participants | Methods, Recruitment and Eligibility section details eligibility criteria, exclusion criteria, participant identification and recruitment methods, and follow-up procedures. |
| **6b** | Matching criteria | Not applicable. This was not a matched cohort study. |
| **7** | Variables | Methods, Data Collection and Clinical Variables and Outcomes and Definitions sections define exposures, risk factors, outcomes, demographic variables, medical history, and pregnancy outcomes. |
| **8** | Data sources/measurement | Methods sections describe collection of clinical data, demographic variables, biological samples, outcome ascertainment, and use of MedSciNet database. |
| **9** | Bias | Standardised protocol, common database, central training and harmonised procedures across sites are described in Multisite study procedures and standardisation. |
| **10** | Study size | Methods, Sample Size and Power Calculations section provides justification and power calculations. |
| **11** | Quantitative variables | Methods, Statistical Analysis and Visualisation section describes summarisation. Continuous variables reported using means, SDs, medians and ranges. No major categorisation except predefined risk classifications and gestational age outcome groups. |
| **12a** | Statistical methods | Methods, Statistical Analysis and Visualisation section describes analytical software and summary methods. As this is a descriptive cohort profile, inferential modelling was not performed. |
| **12b** | Subgroups/interactions | High-risk versus low-risk stratification and outcome subgroup summaries reported; no formal interaction modelling performed. |
| **12c** | Missing data | Methods explicitly describe assessment of completeness and reporting of missing values. |
| **12d** | Loss to follow-up | Addressed in Results (participant flow) and Discussion (limitations related to loss to follow-up). |
| **12e** | Sensitivity analyses | Not applicable. No sensitivity analyses reported for this descriptive cohort profile. |
| **13a** | Participant numbers | Results, Recruitment and Retention section and Figure 1 report numbers consented, retained and analysed. |
| **13** | Reasons for non-participation | Figure 1 and Discussion describe loss to follow-up. |
| **13** | Flow diagram | Included as Figure 1. |
| **14** | Descriptive data | Baseline Participant Characteristics (Table 2), Risk Stratification section and supplementary tables provide participant characteristics. |
| **14b** | Missing data | Missing counts reported in Tables and overall completeness described in Methods and Supplementary Tables. |
| **14c** | Follow-up time | Recruitment and follow-up periods are described. In this case follow-up is at delivery and therefore the summary of averages of gestational age at delivery within the Outcome data is also average follow-up time. |
| **15** | Outcome data | Pregnancy and Delivery Outcomes section and Table 3 report outcome frequencies and gestational age summaries. |
| **16a** | Main results | Not applicable. This manuscript is primarily descriptive and does not report adjusted effect estimates. |
| **16b** | Category boundaries | Outcome categories and risk classifications are clearly defined in Methods. |
| **16c** | Absolute risk translation | Not applicable. No relative risk analyses were performed. |
| **17** | Other analyses | Descriptive analyses of risk-factor overlap, geographic distribution, and sample collection reported in Figures 2-4 and supplementary material. |
| **18** | Key results | Discussion and Conclusions summarise findings relative to study objectives. |
| **19** | Limitations | Dedicated Limitations section discusses enrichment strategy, representativeness, recruitment concentration, ethnicity distribution, and loss to follow-up bias. |
| **20** | Interpretation | Discussion and Conclusions provide interpretation in context of previous INSIGHT findings and study limitations. |
| **21** | Generalisability | Discussed in Limitations section, including high-risk enrichment and predominance of recruitment from London sites. |
| **22** | Funding | Funding declaration provided, including funders and fellowships. |
