# Supplementary material for: Investigation into biomarkers to predict preterm birth (INSIGHT): a longitudinal UK pregnancy cohort for spontaneous preterm birth research
Source: Front Reprod Health. 2026 Sep 30;8:1926506. doi: 10.3389/frph.2026.1926506 (PMC13661963; doi:10.3389/frph.2026.1926506)
Supplement: Supplementary file 2 [file Datasheet1.zip › INSIGHTOverview.V5.html]

Investigation into Biomarkers to Predict Preterm Birth (INSIGHT): a longitudinal UK pregnancy cohort for spontaneous preterm birth research - Publication Script


## Table of contents

- Guide
- Setup
  - Load Packages
  - Load Data
- **Methods**
  - 1. Setting
    - First and Last Visit Recorded
  - 2. Recruitment and Eligibility
    - **Figure 1**: Participant Flow Graph
    - **Supplementary Table A**: Site Specific Participant Counts
  - 4. Data Collection and Clinical Variables
    - **Supplementary Table B**: All Recorded Variables and Completeness
  - 5. Biological Sample Collection and Processing
    - **Table 1**: Specimen Source Summary
- **Results**
  - 2. Baseline Participant Characteristics
    - **Table 2**: Demographic Summary
    - **Supplementary Table C [A and B]**: Demographic Comparison: Sitewise and Lost to Follow-Up
    - **Figure 2**: Geographic Distribution
  - 3. Risk Stratification and Risk Factor Distribution
    - Notes and Definitions
    - **Figure 3**: Risk Factor UpSet Plot
    - **Supplementary Table D**: Risk Factor Table - All
    - **Supplementary Table E**: Risk Factor Table - Individual
  - 4. Pregnancy and Delivery Outcomes
    - **Table 3**: Singleton Outcomes
    - **Supplementary Table F**: Multiples Outcomes
  - 5. Sample Collection
    - **Figure 4**: Specimen Collection
    - **Supplementary Table G**: Sample Collection Counts
    - **Supplementary Table H**: Aliquot Collection Statistics
    - **Figure 5**: Participant Visit Pattern (UpSet Plot)
    - **Supplementary Table I**: Participant Visit Pattern (Table)
    - **Supplementary Table J**: Participants Without Samples
- Software and Versions

# Investigation into Biomarkers to Predict Preterm Birth (INSIGHT): a longitudinal UK pregnancy cohort for spontaneous preterm birth research - Publication Script

 Code

Author

Raphaella Jackson

Modified

September 4, 2026

# Guide

This document presents the code used to produce the statistics, figures, tables, and supplementary material included in the paper. The table of contents on the left can be used to navigate between outputs:  
  
- figures are highlighted in **red**  
- tables in **purple**  
- supplementary tables in **orange**  
- supplementary figures in **blue**

To view the underlying source code, click the Code button in the top-right corner of the document.

# Setup

## Load Packages

```
#Load Packages
library(readxl)
library(tidyverse)
```

```
── Attaching core tidyverse packages ──────────────────────── tidyverse 2.0.0 ──
✔ dplyr     1.2.1     ✔ readr     2.2.0
✔ forcats   1.0.1     ✔ stringr   1.6.0
✔ ggplot2   4.0.3     ✔ tibble    3.3.1
✔ lubridate 1.9.5     ✔ tidyr     1.3.2
✔ purrr     1.2.2     
── Conflicts ────────────────────────────────────────── tidyverse_conflicts() ──
✖ dplyr::filter() masks stats::filter()
✖ dplyr::lag()    masks stats::lag()
ℹ Use the conflicted package (<http://conflicted.r-lib.org/>) to force all conflicts to become errors
```

```
library(consort)
library(flextable)
```

```
Attaching package: 'flextable'

The following object is masked from 'package:purrr':

    compose
```

```
library(gtsummary)
```

```
Attaching package: 'gtsummary'

The following object is masked from 'package:flextable':

    continuous_summary
```

```
library(UpSetR)
library(reshape2)
```

```
Attaching package: 'reshape2'

The following object is masked from 'package:tidyr':

    smiths
```

```
# Packages used but not fully loaded
# library(dlookR)
# library(sf)
```

## Load Data

```
#Clear Data Space (Sanity Clear)
rm(list=ls())

#Load Data
Intake.DF <- read.csv(file = "../Data/Intake.Dataframe.csv",check.names = FALSE)
Outcome.DF <- read.csv(file = "../Data/Outcomes.Dataframe.csv",check.names = FALSE)
Visits.DF <- read.csv(file = "../Data/Visits.Dataframe.csv",check.names = FALSE)
Specimens.DF <- read.csv(file = "../Data/Specimens.Dataframe.csv",check.names = FALSE)
ParticipantCounts.DF <- read.csv(file="../Data/ParticipantCounts.csv",check.names = FALSE)
SpecimenDetails.DF <- read.csv(file="../Data/Specimen_Details.csv",check.names = FALSE)
LTF.DF <- read.csv(file="../Data/LTF.Dataframe.csv",check.names = FALSE)

IMD.Info <- read.csv(file = "../Data/IMDInformation.csv",check.names = FALSE)
```

# **Methods**

## 1. Setting

### First and Last Visit Recorded

```
First_Visit <- min(as.Date(Visits.DF$`Date of visit`,format="%Y-%m-%d"))
Last_Visit <- max(as.Date(Visits.DF$`Date of visit`,format="%Y-%m-%d"))
```

NoteQuick Facts

The first visit of the study was conducted 2013-09-11 .  
The last visit of the study was conducted 2024-06-05 .

## 2. Recruitment and Eligibility

```
#Enrolled/Consented
Total_Enrolled.Count <- sum(colSums(ParticipantCounts.DF[,-1]))

#Exclusions
Lost_to_Follow_Up_Late.Count <- sum(ParticipantCounts.DF$Lost_to_Follow_Up_Late)
Lost_to_Followup_Early.Count <- sum(ParticipantCounts.DF$Lost_to_Follow_Up_Early)
Terminations.Count <- sum(ParticipantCounts.DF$Terminations)

All_Excluded.Count <- Lost_to_Follow_Up_Late.Count + Lost_to_Followup_Early.Count + Terminations.Count

#Retained for Analysis
Analysed.Count <- nrow(Intake.DF)

#Site Counts
GSTT.Count <- Intake.DF %>% filter(Centre %in% "STH") %>% nrow()
PMH.Count <- Intake.DF %>% filter(Centre %in% "PMH") %>% nrow()
MAN.Count <- Intake.DF %>% filter(Centre %in% "MAN") %>% nrow()
WM.Count <- Intake.DF %>% filter(Centre %in% "WM") %>% nrow()

### Flow Plot ###############################################################

#Generate Text
Consented_Box <- paste("Consented (n = ",Total_Enrolled.Count,")",sep = "")
Exclude_Aside <- paste("Excluded (",
                       All_Excluded.Count,
                       "):\n• Lost to Follow-up before Delivery (n = ",
                       Lost_to_Followup_Early.Count,
                       ")\n• Lost to Follow-up at Delivery (n = ",
                       Lost_to_Follow_Up_Late.Count,
                       ")\n• Terminations (n = ",
                       Terminations.Count, 
                       ")\n• Withdrew (n = 0)",
                       sep = "")
Analysed_Box <- paste("Retained for Analysis (n = ",Analysed.Count,")",sep = "")
Arm.GSTT <- paste( "GSTT (n = ",GSTT.Count,")",sep="")
Arm.PMH <- paste( "PMH  (n = ",PMH.Count,")",sep="")
Arm.MAN <- paste( "MAN (n = ",MAN.Count,")",sep="")
Arm.WM <- paste( "WM (n = ",WM.Count,")",sep="")

g <- add_box(,txt = Consented_Box)
g <- add_side_box(g,txt = Exclude_Aside)
g <- add_box(g,txt = Analysed_Box)
g <- add_split(g, txt = c(Arm.GSTT, Arm.PMH,Arm.MAN,Arm.WM))

jpeg("../Stored_Resources/Figures/Plot.Consort.jpeg", width = 10, height = 5, units = "in", res = 300)
g
dev.off()
```

```
quartz_off_screen 
                2
```

```
flow_plot <- g

### Site Specific Table ###############################################################

ParticipantCounts.DF$Consented <- rowSums(ParticipantCounts.DF[,-1])

Out_Table <- ParticipantCounts.DF %>% select(Centre,Consented, Lost_to_Follow_Up_Early,Terminations,Lost_to_Follow_Up_Late,Enrolled) %>% 
    rename(`Lost to Follow-up - Before Delivery` = Lost_to_Follow_Up_Early,
           `Lost to Follow-up - At Delivery` = Lost_to_Follow_Up_Late,
           `Total Retained for Analysis` = Enrolled,
           `Total Consented` = Consented)

Out_Table[5,] <- append("All Sites",colSums(Out_Table[,-1]))

Site_Table <- Out_Table %>% 
  flextable() %>% 
  flextable::vline(j=1) %>% 
  flextable::hline(i=4) %>%
  flextable::line_spacing(space = 1.1, part = "body") %>% 
  flextable::autofit()
```

### **Figure 1**: Participant Flow Graph

```
flow_plot
```

Note**Figure 1**: Participant Flow Diagram

Flow of participants from consent to inclusion in the analysis cohort, with retained participants stratified by recruitment site. “Lost to follow-up before delivery” refers to participants who consented but did not complete any research visits. “Lost to follow-up at delivery” refers to participants for whom basic delivery outcome data, including gestation at delivery and mode of delivery, could not be collected. GSTT denotes Guy’s and St Thomas’ NHS Foundation Trust, London; MAN denotes St Mary’s Hospital, Manchester University NHS Foundation Trust; PMH denotes St Mary’s Hospital, University Hospitals Dorset NHS Foundation Trust; WM denotes West Middlesex University Hospital, Chelsea and Westminster Hospital NHS Foundation Trust. A site-specific breakdown of consented, excluded, and retained participants is provided in Supplementary Table A.

### **Supplementary Table A**: Site Specific Participant Counts

```
Site_Table
```

| Centre | Total Consented | Lost to Follow-up - Before Delivery | Terminations | Lost to Follow-up - At Delivery | Total Retained for Analysis |
| --- | --- | --- | --- | --- | --- |
| MAN | 105 | 1 | 1 | 0 | 103 |
| PMH | 473 | 0 | 4 | 2 | 467 |
| GSTT | 1826 | 12 | 13 | 141 | 1660 |
| WM | 46 | 3 | 0 | 1 | 42 |
| All Sites | 2450 | 16 | 18 | 144 | 2272 |

Note**Supplementary Table A**: Site Specific Participant Counts

Participant recruitment, exclusion, and retention counts by study site, with totals across all sites.

## 4. Data Collection and Clinical Variables

```
### Intake #####################################################################

#Diagnose
Intake.Diagnosis <- dlookr::diagnose(Intake.DF)
```

```
Registered S3 methods overwritten by 'dlookr':
  method          from  
  plot.transform  scales
  print.transform scales
```

```
Intake.Diagnosis.Publish <- Intake.Diagnosis %>% select(variables,missing_count,missing_percent)

#Give Better Names
Intake.Diagnosis.Publish[Intake.Diagnosis.Publish$variables %in% "Centre","variables"] <- "Site"
Intake.Diagnosis.Publish[Intake.Diagnosis.Publish$variables %in% "Final agreed EDD","variables"] <- "Estimated Delivery Date"

#Add Type
Intake.Diagnosis.Publish$Variable_Type <- rep("Intake",nrow(Intake.Diagnosis.Publish))

### Outcomes ###################################################################

#Diagnose
Outcomes.Diagnosis <- dlookr::diagnose(Outcome.DF)
Outcomes.Diagnosis.Publish <- Outcomes.Diagnosis %>% select(variables,missing_count,missing_percent)

#Give Better Names
Outcomes.Diagnosis.Publish$variables <- str_replace(Outcomes.Diagnosis.Publish$variables, "Did she receive","Received")
Outcomes.Diagnosis.Publish$variables <- str_remove(Outcomes.Diagnosis.Publish$variables, "\\?")
Outcomes.Diagnosis.Publish[Outcomes.Diagnosis.Publish$variables %in% "Mag Sulph for fetal neuroprotection","variables"] <- "Received magnesium sulphate for fetal neuroprotection"
Outcomes.Diagnosis.Publish[Outcomes.Diagnosis.Publish$variables %in% "CRP","variables"] <- "Elevated CRP"
Outcomes.Diagnosis.Publish[Outcomes.Diagnosis.Publish$variables %in% "WCC","variables"] <- "Elevated WCC"

Outcomes.Diagnosis.Publish$Variable_Type <- rep("Outcome",nrow(Outcomes.Diagnosis.Publish))

### Combine ####################################################################

VariableSummary <- bind_rows(Intake.Diagnosis.Publish,Outcomes.Diagnosis.Publish)
VariableSummary$missing_percent <- round(VariableSummary$missing_percent,digits=2)
VariableSummary <- VariableSummary %>% rename(`Percent Unreported` = missing_percent,
                                              `Count Unreported` = missing_count,
                                              `Variable Type` = Variable_Type,
                                              `Variable` = variables)
write.csv(VariableSummary,file="../Stored_Resources/Tables/Supplementary.Table.VariableSummary.csv",row.names = FALSE)
```

### **Supplementary Table B**: All Recorded Variables and Completeness

```
DT::datatable(
  VariableSummary, extensions = "Buttons", rownames = FALSE,
  options = list(
    dom = "Bfrtip",
    buttons = list(list(extend = "copy",text = "Copy table")),
    pageLength = 10))
```

Note**Supplementary Table B**: Dataset Completeness

Completeness of recorded intake and outcome variables in the study dataset. **Please Note**:Intake variables are recorded once per participant; outcome variables are recorded once per baby.

## 5. Biological Sample Collection and Processing

```
### Specimen Details to Visualize
Specimen_Details_Table <-
  SpecimenDetails.DF %>%
  flextable() %>%
  set_header_labels(
    values = setNames(
      tools::toTitleCase(gsub("_", " ", names(SpecimenDetails.DF))),
      names(SpecimenDetails.DF)
    )
  ) %>%
  theme_vanilla() %>%
  bg(part = "header", bg = "#2F5597") %>%
  color(part = "header", color = "white") %>%
  bold(part = "header") %>%
  align(part = "header", align = "center") %>%
  align_text_col(align = "left") %>%
  align_nottext_col(align = "right") %>%
  padding(padding = 5, part = "all") %>%
  valign(valign = "center", part = "all") %>%
  autofit() %>%
  set_table_properties(
    layout = "autofit",
    align = "center"
  )

### Save for inclusion into word document (manuscript)

Out_Details <- SpecimenDetails.DF %>%
  flextable() %>% 
  flextable::line_spacing(space = 1.1, part = "body") %>% 
  fontsize(size = 9, part = "all") %>%
  padding(padding = 2, part = "all") %>%
  line_spacing(space = 0.9, part = "all") %>%
  width(j = 1, width = 2.0) %>%
  width(j = 2, width = 4.0) %>%
  set_table_properties(
    layout = "fixed",
    align = "center"
  )

save_as_docx(Out_Details,path="../Stored_Resources/Tables/Table.SpecimenDetails.docx")
```

### **Table 1**: Specimen Source Summary

```
Specimen_Details_Table
```

| Sample Source | Derived Sample | Manuscript Sample Name |
| --- | --- | --- |
| Blood in serum separator tube (SST) | Serum | Serum |
| Blood in tube containing ethylenediaminetetraacetic acid (EDTA) | Plasma | Plasma |
| Blood in tube containing ethylenediaminetetraacetic acid (EDTA) | Buffy coat | Buffy coat |
| Microbiology Swab 1 | Swab smear on glass slide | Microbiology slide |
| Microbiology Swab 1 | Swab suspension in liquid Amies | Swab stored in liquid Amies |
| Microbiology Swab 2 | Swab suspension in TE buffer | Swab stored in TE buffer |
| High vaginal swab | Cervicovaginal fluid | High vaginal fluid |
| High vaginal swab | Cell Pellet | High vaginal cell pellet |
| Low vaginal swab | Cervicovaginal fluid | Low vaginal fluid |
| Low vaginal swab | Cell Pellet | Low vaginal cell pellet |
| Cytobrush | Cytobrush fluid | Cytobrush fluid |
| Cytobrush | Cell Pellet | Cytobrush cell pellet |

Note**Table 1**: Specimen Source Summary

Biological specimen types collected in the INSIGHT study, showing the sample source, the derived sample generated from that source, and the simplified sample name used throughout the manuscript and associated outputs. SST denotes serum separator tube; EDTA denotes ethylenediaminetetraacetic acid; TE denotes Tris-EDTA buffer. Microbiology swabs labelled “1” and “2” indicate two separate swabs collected using the same sampling procedure; the numbering distinguishes their downstream processing or storage purpose rather than a difference in collection method.

# **Results**

## 2. Baseline Participant Characteristics

```
#Compute BMI
Intake.DF$BMI <- Intake.DF$Weight/((Intake.DF$Height/100)^2)

#Give Gestation at Recruitment in Weeks (and fractions thereof) for ease of interpretation
Intake.DF$`Gestation at Recruitment (Weeks)` <- Intake.DF$`Gestation at Recruitment`/7

#Add IMD Information
IMD_Select <- IMD.Info %>% 
                  select(`LSOA code (2011)`,
                         `Index of Multiple Deprivation (IMD) Decile (where 1 is most deprived 10% of LSOAs)`) %>%
                  rename(`Lower super output area` = "LSOA code (2011)")

Intake.DF <- merge(Intake.DF,IMD_Select,by="Lower super output area",all.x = TRUE)

#Correct Site Name for Display
Intake.DF[Intake.DF$Centre %in% "STH","Centre"] <- "GSTT"

gtsummary::theme_gtsummary_compact(set_theme = TRUE, font_size = 10)
```

```
Setting theme "Compact"
```

```
adv <- Intake.DF %>% 
  select(`Age at Registration`,
         `Ethnic Background`,
         BMI,
         `Index of Multiple Deprivation (IMD) Decile (where 1 is most deprived 10% of LSOAs)`,
         `Gestation at Recruitment (Weeks)`,
         Primigravida,
         `Pregnant with multiples`
         ) %>%
  tbl_summary(
    percent="column",
    type = all_continuous() ~ "continuous2",
    statistic = list(
      all_continuous() ~ c(
        "{mean} ({sd})",
        "{median} ({p25}, {p75})",
        "{min}, {max}"
      ),
      all_categorical() ~ "{n} ({p}%)"
    ),
    digits = list(all_continuous() ~ 1),
    missing = "ifany",
    missing_text = "Not Reported",
    missing_stat = "{N_miss} ({p_miss}%)"
  ) %>%
  bold_labels() %>%
  gtsummary::as_flex_table()

demographics_table <- adv %>% 
  flextable::line_spacing(space = 1.1, part = "body") %>% 
  fontsize(size = 9, part = "all") %>%
  padding(padding = 2, part = "all") %>%
  line_spacing(space = 0.9, part = "all") %>%
  width(j = 1, width = 2.0) %>%
  width(j = 2, width = 4.0) %>%
  set_table_properties(
    layout = "fixed",
    align = "center"
  )

save_as_docx(demographics_table,path="../Stored_Resources/Tables/Table.DemographicRisk.docx")

############# Comparative Demographics: By Site #############################################

gtsummary::theme_gtsummary_compact(set_theme = TRUE, font_size = 10)
```

```
Setting theme "Compact"
```

```
adv <- Intake.DF %>% 
  select(Centre,
         `Age at Registration`,
         `Ethnic Background`,
         BMI,
         `Index of Multiple Deprivation (IMD) Decile (where 1 is most deprived 10% of LSOAs)`,
         `Gestation at Recruitment (Weeks)`,
         Primigravida,
         `Pregnant with multiples`,
         `High Risk`
         ) %>%
  tbl_summary(
    by = Centre,
    percent="column",
    type = all_continuous() ~ "continuous2",
    statistic = list(
      all_continuous() ~ c(
        "{mean} ({sd})",
        "{median} ({p25}, {p75})",
        "{min}, {max}"
      ),
      all_categorical() ~ "{n}/{N} ({p}%)"
    ),
    digits = list(all_continuous() ~ 1),
    missing = "ifany",
    missing_text = "Not Reported",
    missing_stat = "{N_miss} ({p_miss}%)"
  ) %>%
  bold_labels() %>%
  gtsummary::as_flex_table()

demographics_table_sitewise <- adv %>% 
  flextable::line_spacing(space = 1.1, part = "body") %>% 
  flextable::add_header_lines(values = "[A] Demographic Comparison - Sitewise") %>%
  flextable::bold(i = 1, bold = TRUE, part = "header") %>%
  flextable::align(i = 1, align = "center", part = "header") %>%
  fontsize(size = 9, part = "all") %>%
  padding(padding = 2, part = "all") %>%
  line_spacing(space = 0.9, part = "all") %>%
  width(j = 1, width = 2.0) %>%
  width(j = 2, width = 4.0) %>%
  set_table_properties(
    layout = "fixed",
    align = "center"
  )

############# Comparative Demographics: Lost to Follow-Up ##################

#Add IMD Information to LTF Dataset
IMD_Select <- IMD.Info %>% 
                  select(`LSOA code (2011)`,
                         `Index of Multiple Deprivation (IMD) Decile (where 1 is most deprived 10% of LSOAs)`) %>%
                  rename(`Lower super output area` = "LSOA code (2011)")
LTF.DF <- merge(LTF.DF,IMD_Select,by="Lower super output area",all.x = TRUE)
LTF.DF$`Age at Registration` <- LTF.DF$`Age at registration`

#Combine LTF Dataset and Analysis Dataset
Full_Set <- bind_rows(LTF.DF,Intake.DF) %>% 
  select(`Age at Registration`,
         `Ethnic Background`,
         `Index of Multiple Deprivation (IMD) Decile (where 1 is most deprived 10% of LSOAs)`,
         `Gestation at Recruitment (Weeks)`,
         `High Risk`,
         LTF_Type) 

Full_Set[is.na(Full_Set$LTF_Type),"LTF_Type"] <- "Analysis Dataset"

LTF_Comparison <- Full_Set %>%
  tbl_summary(
    by= LTF_Type,
    percent="column",
    type = all_continuous() ~ "continuous2",
    statistic = list(
      all_continuous() ~ c(
        "{mean} ({sd})",
        "{median} ({p25}, {p75})",
        "{min}, {max}"
      ),
      all_categorical() ~ "{n}/{N} ({p}%)"
    ),
    digits = list(all_continuous() ~ 1),
    missing = "ifany",
    missing_text = "Not Reported",
    missing_stat = "{N_miss} ({p_miss}%)"
  ) %>%
  bold_labels() %>%
  gtsummary::as_flex_table()

LTF_Comparison <- LTF_Comparison %>% 
  flextable::line_spacing(space = 1.1, part = "body") %>% 
  flextable::add_header_lines(values = "[B] Demographic Comparison - Lost to Follow-Up") %>%
  flextable::bold(i = 1, bold = TRUE, part = "header") %>%
  flextable::align(i = 1, align = "center", part = "header") %>%
  fontsize(size = 9, part = "all") %>%
  padding(padding = 2, part = "all") %>%
  line_spacing(space = 0.9, part = "all") %>%
  width(j = 1, width = 2.0) %>%
  width(j = 2, width = 4.0) %>%
  set_table_properties(
    layout = "fixed",
    align = "center"
  )
```

```
quartz_off_screen 
                2
```

### **Table 2**: Demographic Summary

```
demographics_table
```

| Characteristic | N = 2,2721 |
| --- | --- |
| Age at Registration |  |
| Mean (SD) | 32.8 (5.0) |
| Median (Q1, Q3) | 33.0 (30.0, 36.0) |
| Min, Max | 16.0, 52.0 |
| Ethnic Background |  |
| African | 291 (13%) |
| Bangladeshi | 7 (0.3%) |
| Black Caribbean | 110 (5.1%) |
| East Asian | 50 (2.3%) |
| European | 1,474 (68%) |
| Indian | 47 (2.2%) |
| Middle Eastern | 10 (0.5%) |
| Other | 116 (5.4%) |
| Pakistani | 21 (1.0%) |
| South East Asian | 38 (1.8%) |
| Not Reported | 108 (4.8%) |
| BMI |  |
| Mean (SD) | 25.6 (5.4) |
| Median (Q1, Q3) | 24.4 (21.8, 28.3) |
| Min, Max | 12.9, 52.3 |
| Not Reported | 11 (0.5%) |
| Index of Multiple Deprivation (IMD) Decile (where 1 is most deprived 10% of LSOAs) |  |
| Mean (SD) | 4.8 (2.4) |
| Median (Q1, Q3) | 4.0 (3.0, 6.0) |
| Min, Max | 1.0, 10.0 |
| Not Reported | 102 (4.5%) |
| Gestation at Recruitment (Weeks) |  |
| Mean (SD) | 13.6 (2.1) |
| Median (Q1, Q3) | 13.0 (12.3, 14.6) |
| Min, Max | 8.0, 25.7 |
| Primigravida | 633 (28%) |
| Not Reported | 6 (0.3%) |
| Pregnant with multiples | 88 (3.9%) |
|  |  |
| --- | --- |
| 1n (%) | |

Note**Table 2**: Demographic Summary

Sociodemographic, anthropometric, pregnancy, and recruitment characteristics of participants included in the INSIGHT study cohort. Percentages are calculated using the full participant cohort (n=2272) as the denominator unless otherwise indicated. Ethnicity was self-reported and coded using categories informed by Office for National Statistics (ONS) classifications defined at study outset. For reporting, legacy category labels were updated to contemporary terminology: “AfroCaribbean” is reported as “Black Caribbean” and “Far East Asian” as “East Asian”; the underlying participant classifications were unchanged. Gestation at Recruitment reflects final dating following ultrasound. Where early ultrasound redating revised the estimated gestation, the recorded value at recruitment may fall below the 10+0 week enrolment threshold, accounting for the minimum of 8.0 weeks shown.

### **Supplementary Table C [A and B]**: Demographic Comparison: Sitewise and Lost to Follow-Up

```
demographics_table_sitewise
```

| [A] Demographic Comparison - Sitewise | | | | |
| --- | --- | --- | --- | --- |
| Characteristic | GSTT  N = 1,6601 | MAN  N = 1031 | PMH  N = 4671 | WM  N = 421 |
| Age at Registration |  |  |  |  |
| Mean (SD) | 33.2 (4.9) | 31.3 (4.6) | 31.5 (5.0) | 33.1 (5.1) |
| Median (Q1, Q3) | 33.0 (30.0, 36.0) | 31.0 (28.0, 34.0) | 32.0 (28.0, 35.0) | 33.5 (30.0, 36.0) |
| Min, Max | 16.0, 52.0 | 22.0, 43.0 | 17.0, 49.0 | 19.0, 43.0 |
| Ethnic Background |  |  |  |  |
| African | 287/1,552 (18%) | 3/103 (2.9%) | 0/467 (0%) | 1/42 (2.4%) |
| Bangladeshi | 6/1,552 (0.4%) | 0/103 (0%) | 0/467 (0%) | 1/42 (2.4%) |
| Black Caribbean | 105/1,552 (6.8%) | 2/103 (1.9%) | 2/467 (0.4%) | 1/42 (2.4%) |
| East Asian | 47/1,552 (3.0%) | 0/103 (0%) | 2/467 (0.4%) | 1/42 (2.4%) |
| European | 902/1,552 (58%) | 89/103 (86%) | 454/467 (97%) | 29/42 (69%) |
| Indian | 40/1,552 (2.6%) | 1/103 (1.0%) | 1/467 (0.2%) | 5/42 (12%) |
| Middle Eastern | 8/1,552 (0.5%) | 1/103 (1.0%) | 0/467 (0%) | 1/42 (2.4%) |
| Other | 108/1,552 (7.0%) | 2/103 (1.9%) | 6/467 (1.3%) | 0/42 (0%) |
| Pakistani | 16/1,552 (1.0%) | 3/103 (2.9%) | 0/467 (0%) | 2/42 (4.8%) |
| South East Asian | 33/1,552 (2.1%) | 2/103 (1.9%) | 2/467 (0.4%) | 1/42 (2.4%) |
| Not Reported | 108 (6.5%) | 0 (0%) | 0 (0%) | 0 (0%) |
| BMI |  |  |  |  |
| Mean (SD) | 25.6 (5.5) | 25.8 (5.0) | 25.9 (5.1) | 24.7 (3.8) |
| Median (Q1, Q3) | 24.2 (21.6, 28.3) | 24.7 (22.2, 28.7) | 25.0 (22.3, 28.3) | 23.7 (22.3, 26.0) |
| Min, Max | 15.9, 52.1 | 17.1, 42.7 | 12.9, 52.3 | 19.5, 37.2 |
| Not Reported | 11 (0.7%) | 0 (0%) | 0 (0%) | 0 (0%) |
| Index of Multiple Deprivation (IMD) Decile (where 1 is most deprived 10% of LSOAs) |  |  |  |  |
| Mean (SD) | 4.3 (2.1) | 4.0 (2.7) | 6.3 (2.4) | 6.1 (2.5) |
| Median (Q1, Q3) | 4.0 (3.0, 5.0) | 4.0 (1.0, 6.0) | 7.0 (5.0, 8.0) | 6.0 (4.0, 9.0) |
| Min, Max | 1.0, 10.0 | 1.0, 10.0 | 1.0, 10.0 | 1.0, 10.0 |
| Not Reported | 75 (4.5%) | 3 (2.9%) | 22 (4.7%) | 2 (4.8%) |
| Gestation at Recruitment (Weeks) |  |  |  |  |
| Mean (SD) | 13.5 (2.2) | 13.0 (1.1) | 14.2 (1.9) | 14.2 (1.1) |
| Median (Q1, Q3) | 12.9 (12.3, 14.1) | 12.9 (12.6, 13.4) | 14.0 (12.7, 15.6) | 14.1 (13.6, 15.0) |
| Min, Max | 8.0, 25.7 | 10.3, 18.7 | 8.0, 23.3 | 11.9, 15.9 |
| Primigravida | 487/1,655 (29%) | 29/103 (28%) | 109/466 (23%) | 8/42 (19%) |
| Not Reported | 5 (0.3%) | 0 (0%) | 1 (0.2%) | 0 (0%) |
| Pregnant with multiples | 29/1,660 (1.7%) | 0/103 (0%) | 58/467 (12%) | 1/42 (2.4%) |
| High Risk | 830/1,660 (50%) | 38/103 (37%) | 413/467 (88%) | 36/42 (86%) |
|  |  |  |  |  |
| --- | --- | --- | --- | --- |
| 1n/N (%) | | | | |

```
LTF_Comparison
```

| [B] Demographic Comparison - Lost to Follow-Up | | | |
| --- | --- | --- | --- |
| Characteristic | Analysis Dataset  N = 2,2721 | Lost to Follow-Up After Delivery  N = 1441 | Lost to Follow-Up Before Delivery  N = 161 |
| Age at Registration |  |  |  |
| Mean (SD) | 32.8 (5.0) | 32.5 (4.7) | 32.0 (3.7) |
| Median (Q1, Q3) | 33.0 (30.0, 36.0) | 32.5 (30.0, 36.0) | 32.0 (30.0, 33.5) |
| Min, Max | 16.0, 52.0 | 18.0, 46.0 | 25.0, 41.0 |
| Ethnic Background |  |  |  |
| African | 291/2,164 (13%) | 19/133 (14%) | 1/10 (10%) |
| Bangladeshi | 7/2,164 (0.3%) | 0/133 (0%) | 0/10 (0%) |
| Black Caribbean | 110/2,164 (5.1%) | 7/133 (5.3%) | 0/10 (0%) |
| East Asian | 50/2,164 (2.3%) | 2/133 (1.5%) | 0/10 (0%) |
| European | 1,474/2,164 (68%) | 81/133 (61%) | 9/10 (90%) |
| Indian | 47/2,164 (2.2%) | 7/133 (5.3%) | 0/10 (0%) |
| Middle Eastern | 10/2,164 (0.5%) | 2/133 (1.5%) | 0/10 (0%) |
| Other | 116/2,164 (5.4%) | 12/133 (9.0%) | 0/10 (0%) |
| Pakistani | 21/2,164 (1.0%) | 1/133 (0.8%) | 0/10 (0%) |
| South East Asian | 38/2,164 (1.8%) | 2/133 (1.5%) | 0/10 (0%) |
| Not Reported | 108 (4.8%) | 11 (7.6%) | 6 (38%) |
| Index of Multiple Deprivation (IMD) Decile (where 1 is most deprived 10% of LSOAs) |  |  |  |
| Mean (SD) | 4.8 (2.4) | 4.9 (2.5) | 4.5 (2.2) |
| Median (Q1, Q3) | 4.0 (3.0, 6.0) | 4.0 (3.0, 6.0) | 4.0 (3.0, 5.0) |
| Min, Max | 1.0, 10.0 | 1.0, 10.0 | 1.0, 9.0 |
| Not Reported | 102 (4.5%) | 9 (6.3%) | 0 (0%) |
| Gestation at Recruitment (Weeks) |  |  |  |
| Mean (SD) | 13.6 (2.1) | 13.7 (2.6) | 13.6 (2.1) |
| Median (Q1, Q3) | 13.0 (12.3, 14.6) | 12.9 (12.1, 14.3) | 13.1 (12.1, 13.9) |
| Min, Max | 8.0, 25.7 | 10.3, 24.1 | 10.9, 18.7 |
| High Risk | 1,317/2,272 (58%) | 62/144 (43%) | 6/16 (38%) |
|  |  |  |  |
| --- | --- | --- | --- |
| 1n/N (%) | | | |

Note**Supplementary Table C**: Demographic Comparison: Lost to Follow-Up

A comparison of basic demographic characteristics of **[A]** participants retained within the analysis cohort broken down by site **[B]** all consented participants broken down by whether they were lost to follow-up early/late or retained in the analysis cohort. Ethnicity was self-reported and coded using categories informed by Office for National Statistics (ONS) classifications defined at study outset. For reporting, legacy category labels were updated to contemporary terminology: “AfroCaribbean” is reported as “Black Caribbean” and “Far East Asian” as “East Asian”; the underlying participant classifications were unchanged. High risk indicates the participant’s final risk status determination based on the criteria listed under Recruitment and Eligibility, based on all the information available at the conclusion of the study.

### **Figure 2**: Geographic Distribution

```
Map
```

Note**Figure 2**:Map of Participant LSOAs

Map showing lower layer super output areas (LSOAs) represented by participants in the INSIGHT study cohort. LSOAs containing at least one participant are shaded navy; all other English LSOAs are shaded grey. LSOA denotes lower layer super output area, a small-area geographic unit used for reporting population statistics in England.

## 3. Risk Stratification and Risk Factor Distribution

```
#The column for cervical length is stored as <25mm even though it should be ≤25mm because the ≤ symbol can't be stored in the database and isn't read in properly by R. As a result we fix the name after it is loaded.

High_Risk_Participants <- Intake.DF %>% filter(`High Risk` %in% "Yes")
High_Risk_Participants.Count <- nrow(High_Risk_Participants)

Cervix_Measured <- Intake.DF %>% filter(!`Cervical length <25mm (19-24+6 weeks)` %in% "Not Measured")
Cervix_Measured.Count <- nrow(Cervix_Measured)

HighRisk.Table <- High_Risk_Participants %>% select(`Participant ID`,
                                                    `Previous spontaneous PTB (<37 weeks)`,
                                                    `Previous midtrimester loss (16-23+6 weeks)`,
                                                    `Previous PPROM (<37 weeks)`,
                                                    `Cervical length <25mm (19-24+6 weeks)`,
                                                    `Previous cervical surgery (e.g. LLETZ, Cone)`,
                                                    `Pregnant with multiples`,
                                                    `Uterine abnormality`)


colnames(HighRisk.Table) <- c("Participant ID",
                              "Previous sPTB",
                              "Previous MTL",
                              "Previous PPROM",
                              "Cervical Length ≤ 25",
                              "Cervical History",
                              "Multiple Pregnancy",
                              "Uterine Variant")

Risk_Counts_All.df <- HighRisk.Table %>%
  count(across(all_of(colnames(HighRisk.Table[,-1]))), name = "Number of Participants")

HighRisk.Table$`*Cervix Not Measured` <- rep("No",nrow(HighRisk.Table))
HighRisk.Table[!HighRisk.Table$`Participant ID` %in% Cervix_Measured$`Participant ID`, "*Cervix Not Measured"] <- "Yes"

CombMat <- HighRisk.Table[,-1]
CombMat[CombMat=="Yes"] <- 1
CombMat[CombMat=="No"] <- 0
CombMat[CombMat=="Not Measured"] <- 0
CombMat <- CombMat %>% mutate_if(is.character, as.numeric)
CombMat <- CombMat[,colSums(CombMat)>0]
CombMat <- data.frame(CombMat,check.names = FALSE)

Risk_Counts_Individual.df <- CombMat %>%
  summarise(across(everything(), ~ sum(.x == 1, na.rm = TRUE))) %>%
  tidyr::pivot_longer(everything(),names_to = "Risk Factor",values_to = "Number of Participants")

UpsetPlot <- UpSetR::upset(CombMat,nsets = 25, text.scale = c(1.5, 1.5, 1.3, 1.3, 1.4, 1.1))
```

```
Warning: `aes_string()` was deprecated in ggplot2 3.0.0.
ℹ Please use tidy evaluation idioms with `aes()`.
ℹ See also `vignette("ggplot2-in-packages")` for more information.
ℹ The deprecated feature was likely used in the UpSetR package.
  Please report the issue at <https://github.com/hms-dbmi/UpSetR/issues>.
```

```
Warning: Using `size` aesthetic for lines was deprecated in ggplot2 3.4.0.
ℹ Please use `linewidth` instead.
ℹ The deprecated feature was likely used in the UpSetR package.
  Please report the issue at <https://github.com/hms-dbmi/UpSetR/issues>.
```

```
Warning: The `size` argument of `element_line()` is deprecated as of ggplot2 3.4.0.
ℹ Please use the `linewidth` argument instead.
ℹ The deprecated feature was likely used in the UpSetR package.
  Please report the issue at <https://github.com/hms-dbmi/UpSetR/issues>.
```

```
jpeg("../Stored_Resources/Figures/Plot.UPSETRisk.jpeg", width = 9, height = 5, units = "in", res = 300)
UpsetPlot
dev.off()
```

```
quartz_off_screen 
                2
```

### Notes and Definitions

NoteRisk Profiles of Participants

Participants with varying risk profiles for preterm birth were recruited.  
Here we consider participants to be at high risk of preterm birth if they fulfilled one or more of the following criteria which were specified as being high risk criteria for the INSIGHT study.

• [Previous PTB] Previous preterm birth or mid-trimester loss between 16+0 and 36+6 weeks’ gestation.  
• [Previous PPROM] Previous preterm prelabour rupture of the fetal membranes (PPROM) at ≤ 36+6 weeks’ gestation.  
• [Cervical Length ≤25] Short cervical length (≤ 25 mm) on ultrasound between 18+0 and 24+0 weeks’ gestation.  
• [Cervical History] Previous cervical procedure to treat abnormal smears (large loop excision, laser conisation, cold knife conisation, or radical diathermy).  
• [Multiple Pregnancy] Multiple pregnancy  
• [Uterine Variant] Known Müllerian Abnormalities

NoteRisk Profiles of Participants

A total of 1317 high risk participants were recruited.

### **Figure 3**: Risk Factor UpSet Plot

Important

Not having a measurement of the cervix is not considered a risk factor. However, we identify participants who were classified as high risk who did not have their cervix measured as it is not known whether they may have had a short cervix or not.

```
UpsetPlot
```

Note**Figure 3**: Risk Factor UpSet Plot

UpSet plot showing the individual and overlapping risk factors used to classify participants as being at high risk of preterm birth. Set size indicates the number of participants with each risk factor irrespective of other co-occurring risk factors; intersection size indicates the number of participants with each specific combination of risk factors. Solid black circles indicate risk factors present in each group, and connecting black lines indicate combinations involving more than one risk factor. sPTB denotes spontaneous preterm birth; MTL denotes mid-trimester loss between 16+0 and 23+6 weeks’ gestation (mid-trimester loss between 14+0 and 15+6 weeks’ gestation is not included in this criterion); PPROM denotes preterm prelabour rupture of membranes. Cervical history refers to previous cervical procedures. All risk factors are fully defined in the Recruitment and Eligibility section. The reported frequencies are the final, updated risk status of participants, in cases where participants had a cervical measurement ≤ 25 mm on ultrasound between 18+0 and 24+0 weeks’ gestation which occurred after their enrolment visit.

### **Supplementary Table D**: Risk Factor Table - All

```
DT::datatable(
  Risk_Counts_All.df, extensions = "Buttons", rownames = FALSE,
  options = list(
    dom = "Bfrtip",
    buttons = list(list(extend = "copy",text = "Copy table")),
    pageLength = 10))
```

Note**Supplementary Table D**: Risk Factors (Intersecting)

Counts of distinct combinations of preterm birth risk factors among high-risk participants, corresponding to the intersection-size bars in Figure 3.

### **Supplementary Table E**: Risk Factor Table - Individual

```
DT::datatable(
  Risk_Counts_Individual.df, extensions = "Buttons", rownames = FALSE,
  options = list(
    dom = "Bfrtip",
    buttons = list(list(extend = "copy",text = "Copy table")),
    pageLength = 10))
```

Note**Supplementary Table E**: Risk Factors (Independent)

Counts of participants with each individual preterm birth risk factor, corresponding to the set-size bars in Figure 3.

## 4. Pregnancy and Delivery Outcomes

```
# Add Gestation at Delivery in Weeks plus Fraction of Weeks
Outcome.DF$`Gestation at Delivery (Weeks)` <- (Outcome.DF$`Gestation at delivery (w)`*7+Outcome.DF$`Gestation at delivery (d)`)/7

# Add Risk and Multiple Information to Table
T1 <- nrow(Outcome.DF)
Tmp <- Intake.DF %>% select(`Participant ID`,`High Risk`,`Pregnant with multiples`)
Outcome.DF <- merge(Outcome.DF,Tmp,by="Participant ID",all = TRUE)
T2 <- nrow(Outcome.DF)
stopifnot(T1 == T2)

#Make the formatting a bit nicer on the categories
Outcome.DF$PTB_Type_Formatted <- Outcome.DF %>% mutate(
  LS = case_when(
    PTB_Type %in% "Term"                       ~ "Term",
    PTB_Type %in% "IPTB"                       ~ "iPTB",
    PTB_Type %in% "SPTB"                       ~ "sPTB",
    PTB_Type %in% "MidTrimesterLoss_24"        ~ "Mid-trimester Loss",
    PTB_Type %in% "Miscarriage_14"             ~ "Miscarriage",
    TRUE                                       ~ "NA")) %>%
  pull(LS)

Outcome.DF$PTB_Type_Formatted <- factor(Outcome.DF$PTB_Type_Formatted ,
                                        levels = c("Term","iPTB","sPTB","Mid-trimester Loss","Miscarriage"))

#Group MTL and Miscarriage outcome to not be repetitive
Outcome.DF[Outcome.DF$PTB_Type_Formatted %in% c("Mid-trimester Loss","Miscarriage"),"Pregnancy Outcome"] <- "Loss Before 24 Weeks"
Outcome.DF$`Pregnancy Outcome` <- factor(Outcome.DF$`Pregnancy Outcome`, levels = c("Live birth","Stillbirth","Loss Before 24 Weeks"))

#Clarify the Stillbirth and Miscarriage are not applicable for NICU admission or inpatient nights
Outcome.DF <- Outcome.DF %>%
  mutate(Neonatal_Outcome_Applicable = !(PTB_Type_Formatted %in% c("Miscarriage","Mid-trimester Loss") |
                                         `Pregnancy Outcome` %in% "Stillbirth"),
    `Admission to SCBU or NICU` = if_else(Neonatal_Outcome_Applicable,
                                          `Admission to SCBU or NICU`,
      "Not Applicable"))

##### Singleton Outcomes #######################################################

# Singletons
Singles.Table_Values <- Outcome.DF %>%
  filter(`Pregnant with multiples` %in% "No") %>%
  mutate(
    Inpatient_Nights_Raw = as.character(`Number of inpatient nights`),
    NICU_Raw = as.character(`Admission to SCBU or NICU`),

    Neonatal_Applicable = !(
      Inpatient_Nights_Raw %in% "Not Applicable" |
      NICU_Raw %in% "Not Applicable"
    ),

    `Number of inpatient nights` = suppressWarnings(as.numeric(case_when(
      Inpatient_Nights_Raw %in% c("Not Applicable", "Not Reported", "NA", "") ~ NA_character_,
      TRUE ~ Inpatient_Nights_Raw
    ))),

    `Admission to SCBU or NICU` = case_when(
      NICU_Raw %in% c("Not Reported", "NA", "") ~ NA_character_,
      TRUE ~ NICU_Raw
    )
  )

#Outcomes - All Births
singleton_outcomes_tbl <- Singles.Table_Values %>%
  select(
    `High Risk`,
    `Pregnancy Outcome`,
    `Gestation at Delivery (Weeks)`,
    PTB_Type_Formatted
  ) %>%
  tbl_summary(
    by = PTB_Type_Formatted,
    percent = "column",
    type = all_continuous() ~ "continuous2",
    statistic = list(
      all_continuous() ~ c(
        "{mean} ({sd})",
        "{median} ({p25}, {p75})",
        "{min}, {max}"
      ),
      all_categorical() ~ "{n}/{N} ({p}%)"
    ),
    digits = list(all_continuous() ~ 1),
    missing = "ifany",
    missing_text = "Not Reported",
    missing_stat = "{N_miss}/{N_obs} ({p_miss}%)"
  ) %>%
  add_overall(last = TRUE) %>%
  bold_labels() %>%
  modify_header(
    label = "",
    all_stat_cols() ~ "**{level}**, N = {n} ({style_percent(p)}%)"
  ) %>%
  gtsummary::as_flex_table()

singletons_outcomes <- singleton_outcomes_tbl %>%
  flextable::vline(j = 1) %>%
  flextable::vline(j = 6) %>%
  flextable::add_header_lines(values = "[A] Pregnancy Outcomes - Singletons") %>%
  flextable::bold(i = 1, bold = TRUE, part = "header") %>%
  flextable::align(i = 1, align = "center", part = "header") %>%
  flextable::line_spacing(space = 1.1, part = "body") %>%
  flextable::autofit()

#Outcomes - Neonatal Care (Where applicable)

neonatal_outcomes_tbl <- Singles.Table_Values %>%
  filter(Neonatal_Applicable) %>%
  mutate(PTB_Type_Formatted = droplevels(PTB_Type_Formatted)) %>%
  select(
    `Admission to SCBU or NICU`,
    `Number of inpatient nights`,
    PTB_Type_Formatted
  ) %>%
  tbl_summary(
    by = PTB_Type_Formatted,
    percent = "column",
    type = all_continuous() ~ "continuous2",
    statistic = list(
      all_continuous() ~ c(
        "{mean} ({sd})",
        "{median} ({p25}, {p75})",
        "{min}, {max}"
      ),
      all_categorical() ~ "{n}/{N} ({p}%)"
    ),
    digits = list(all_continuous() ~ 1),
    missing = "ifany",
    missing_text = "Not Reported",
    missing_stat = "{N_miss}/{N_obs} ({p_miss}%)"
  ) %>%
  add_overall(last = TRUE) %>%
  bold_labels() %>%
  modify_header(
    label = "",
    all_stat_cols() ~ "**{level}**, N = {n} ({style_percent(p)}%)"
  ) %>%
  gtsummary::as_flex_table()

singletons_neonatal_outcomes <- neonatal_outcomes_tbl %>%
  flextable::vline(j = 1) %>%
  flextable::vline(j = 4) %>%
  flextable::add_header_lines(values = "[B] Neonatal Outcomes - Singletons") %>%
  flextable::bold(i = 1, bold = TRUE, part = "header") %>%
  flextable::align(i = 1, align = "center", part = "header") %>%
  flextable::line_spacing(space = 1.1, part = "body") %>%
  flextable::autofit()

save_as_docx(singletons_outcomes,path="../Stored_Resources/Tables/Table.Singletons.Outcomes.docx")
save_as_docx(singletons_neonatal_outcomes,path="../Stored_Resources/Tables/Table.Singletons.NeoOutcomes.docx")

##### Multiples Outcomes #######################################################

# Multplies
Multiples.Table_Values <- Outcome.DF %>%
  filter(`Pregnant with multiples` %in% "Yes") %>%
  mutate(
    Inpatient_Nights_Raw = as.character(`Number of inpatient nights`),
    NICU_Raw = as.character(`Admission to SCBU or NICU`),

    Neonatal_Applicable = !(
      Inpatient_Nights_Raw %in% "Not Applicable" |
      NICU_Raw %in% "Not Applicable"
    ),

    `Number of inpatient nights` = suppressWarnings(as.numeric(case_when(
      Inpatient_Nights_Raw %in% c("Not Applicable", "Not Reported", "NA", "") ~ NA_character_,
      TRUE ~ Inpatient_Nights_Raw
    ))),

    `Admission to SCBU or NICU` = case_when(
      NICU_Raw %in% c("Not Reported", "NA", "") ~ NA_character_,
      TRUE ~ NICU_Raw
    )
  )

#Outcomes - All Births
multiples_outcomes_tbl <- Multiples.Table_Values %>%
  select(
    `High Risk`,
    `Pregnancy Outcome`,
    `Gestation at Delivery (Weeks)`,
    PTB_Type_Formatted
  ) %>%
  mutate(PTB_Type_Formatted = droplevels(PTB_Type_Formatted)) %>%
  tbl_summary(
    by = PTB_Type_Formatted,
    percent = "column",
    type = all_continuous() ~ "continuous2",
    statistic = list(
      all_continuous() ~ c(
        "{mean} ({sd})",
        "{median} ({p25}, {p75})",
        "{min}, {max}"
      ),
      all_categorical() ~ "{n}/{N} ({p}%)"
    ),
    digits = list(all_continuous() ~ 1),
    missing = "ifany",
    missing_text = "Not Reported",
    missing_stat = "{N_miss}/{N_obs} ({p_miss}%)"
  ) %>%
  add_overall(last = TRUE) %>%
  bold_labels() %>%
  modify_header(
    label = "",
    all_stat_cols() ~ "**{level}**, N = {n} ({style_percent(p)}%)"
  ) %>%
  gtsummary::as_flex_table()

multiples_outcomes <- multiples_outcomes_tbl %>%
  flextable::vline(j = 1) %>%
  flextable::vline(j = 6) %>%
  flextable::add_header_lines(values = "[A] Pregnancy Outcomes - Multiples") %>%
  flextable::bold(i = 1, bold = TRUE, part = "header") %>%
  flextable::align(i = 1, align = "center", part = "header") %>%
  flextable::line_spacing(space = 1.1, part = "body") %>%
  flextable::autofit()

#Outcomes - Neonatal Care (Where applicable)
multiples_outcomes_tbl <- Multiples.Table_Values %>%
  filter(Neonatal_Applicable) %>%
  mutate(PTB_Type_Formatted = droplevels(PTB_Type_Formatted)) %>%
  select(
    `Admission to SCBU or NICU`,
    `Number of inpatient nights`,
    PTB_Type_Formatted
  ) %>%
  tbl_summary(
    by = PTB_Type_Formatted,
    percent = "column",
    type = all_continuous() ~ "continuous2",
    statistic = list(
      all_continuous() ~ c(
        "{mean} ({sd})",
        "{median} ({p25}, {p75})",
        "{min}, {max}"
      ),
      all_categorical() ~ "{n}/{N} ({p}%)"
    ),
    digits = list(all_continuous() ~ 1),
    missing = "ifany",
    missing_text = "Not Reported",
    missing_stat = "{N_miss}/{N_obs} ({p_miss}%)"
  ) %>%
  add_overall(last = TRUE) %>%
  bold_labels() %>%
  modify_header(
    label = "",
    all_stat_cols() ~ "**{level}**, N = {n} ({style_percent(p)}%)"
  ) %>%
  gtsummary::as_flex_table()

multiples_neonatal_outcomes <- multiples_outcomes_tbl %>%
  flextable::vline(j = 1) %>%
  flextable::vline(j = 4) %>%
  flextable::add_header_lines(values = "[B] Neonatal Outcomes - Multiples") %>%
  flextable::bold(i = 1, bold = TRUE, part = "header") %>%
  flextable::align(i = 1, align = "center", part = "header") %>%
  flextable::line_spacing(space = 1.1, part = "body") %>%
  flextable::autofit()

save_as_docx(multiples_outcomes,path="../Stored_Resources/Tables/Table.Multiples.Outcomes.docx")
save_as_docx(multiples_neonatal_outcomes,path="../Stored_Resources/Tables/Table.Multiples.NeoOutcomes.docx")
```

### **Table 3**: Singleton Outcomes

```
singletons_outcomes
```

| [A] Pregnancy Outcomes - Singletons | | | | | | |
| --- | --- | --- | --- | --- | --- | --- |
|  | Term, N = 1899 (87%)1 | iPTB, N = 64 (2.9%)1 | sPTB, N = 184 (8.4%)1 | Mid-trimester Loss, N = 36 (1.6%)1 | Miscarriage, N = 1 (<0.1%)1 | Overall, N = 2184 (100%)1 |
| High Risk | 992/1,899 (52%) | 43/64 (67%) | 160/184 (87%) | 34/36 (94%) | 0/1 (0%) | 1,229/2,184 (56%) |
| Pregnancy Outcome |  |  |  |  |  |  |
| Live birth | 1,897/1,899 (100%) | 60/64 (94%) | 176/184 (96%) | 0/36 (0%) | 0/1 (0%) | 2,133/2,184 (98%) |
| Stillbirth | 2/1,899 (0.1%) | 4/64 (6.3%) | 8/184 (4.3%) | 0/36 (0%) | 0/1 (0%) | 14/2,184 (0.6%) |
| Loss Before 24 Weeks | 0/1,899 (0%) | 0/64 (0%) | 0/184 (0%) | 36/36 (100%) | 1/1 (100%) | 37/2,184 (1.7%) |
| Gestation at Delivery (Weeks) |  |  |  |  |  |  |
| Mean (SD) | 39.6 (1.2) | 34.8 (2.2) | 32.9 (4.2) | 18.5 (2.1) | 12.1 (NA) | 38.5 (3.7) |
| Median (Q1, Q3) | 39.6 (38.7, 40.6) | 35.4 (34.2, 36.3) | 34.6 (31.1, 36.2) | 18.4 (17.0, 19.9) | 12.1 (12.1, 12.1) | 39.3 (38.1, 40.3) |
| Min, Max | 37.0, 42.6 | 27.7, 36.9 | 20.6, 36.9 | 14.3, 23.6 | 12.1, 12.1 | 12.1, 42.6 |
|  |  |  |  |  |  |  |
| --- | --- | --- | --- | --- | --- | --- |
| 1n/N (%) | | | | | | |

```
singletons_neonatal_outcomes
```

| [B] Neonatal Outcomes - Singletons | | | | |
| --- | --- | --- | --- | --- |
|  | Term, N = 1897 (89%)1 | iPTB, N = 60 (2.8%)1 | sPTB, N = 176 (8.3%)1 | Overall, N = 2133 (100%)1 |
| Admission to SCBU or NICU | 57/1,896 (3.0%) | 32/60 (53%) | 93/176 (53%) | 182/2,132 (8.5%) |
| Not Reported | 1/1,897 (<0.1%) | 0/60 (0%) | 0/176 (0%) | 1/2,133 (<0.1%) |
| Number of inpatient nights |  |  |  |  |
| Mean (SD) | 1.9 (2.7) | 12.6 (17.2) | 22.5 (48.7) | 3.7 (14.9) |
| Median (Q1, Q3) | 1.0 (1.0, 2.0) | 7.0 (4.0, 12.5) | 7.0 (4.0, 15.0) | 2.0 (1.0, 3.0) |
| Min, Max | 0.0, 63.0 | 1.0, 94.0 | 1.0, 395.0 | 0.0, 395.0 |
| Not Reported | 69/1,897 (3.6%) | 4/60 (6.7%) | 23/176 (13%) | 96/2,133 (4.5%) |
|  |  |  |  |  |
| --- | --- | --- | --- | --- |
| 1n/N (%) | | | | |

Note**Table 3**: Pregnancy and neonatal outcomes among participants with singleton pregnancies

Outcomes are summarised for participants with singleton pregnancies. Table 3A presents pregnancy outcomes, and Table 3B presents neonatal outcomes. Values are presented as n (%) unless otherwise stated. Percentages in Table 3A are calculated using the number of singleton pregnancies within each outcome group as the denominator; for singleton pregnancies, this is equivalent to the number of participants. Percentages in Table 3B are calculated using the number of singleton live births within each outcome group as the denominator; miscarriages, stillbirths, and mid-trimester losses are excluded from these denominators because admission to SCBU or NICU and inpatient neonatal stay are not applicable to these outcomes, while applicable outcomes not reported are retained as “Not Reported”. Number of inpatient nights refers to the number of nights the infant remained admitted to hospital after birth. Term birth was defined as delivery at ≥37+0 weeks’ gestation. Spontaneous preterm birth (sPTB) was defined as delivery before 37+0 weeks’ gestation following spontaneous onset of labour or preterm prelabour rupture of membranes. Iatrogenic preterm birth (iPTB) was defined as delivery before 37+0 weeks’ gestation following medical intervention, including induction of labour or prelabour caesarean birth for maternal or fetal indications. Mid-trimester loss was defined as pregnancy loss between 14+0 and 23+6 weeks’ gestation. Stillbirth was defined as birth without signs of life at or beyond 24+0 weeks’ gestation. Miscarriage was defined as pregnancy loss before 14+0 weeks’ gestation with known outcome details; missed miscarriages are not included in this category. SCBU denotes Special Care Baby Unit; NICU denotes Neonatal Intensive Care Unit.

### **Supplementary Table F**: Multiples Outcomes

```
multiples_outcomes
```

| [A] Pregnancy Outcomes - Multiples | | | | | |
| --- | --- | --- | --- | --- | --- |
|  | Term, N = 74 (42%)1 | iPTB, N = 52 (30%)1 | sPTB, N = 43 (25%)1 | Mid-trimester Loss, N = 6 (3.4%)1 | Overall, N = 175 (100%)1 |
| High Risk |  |  |  |  |  |
| Yes | 74/74 (100%) | 52/52 (100%) | 43/43 (100%) | 6/6 (100%) | 175/175 (100%) |
| Pregnancy Outcome |  |  |  |  |  |
| Live birth | 74/74 (100%) | 52/52 (100%) | 42/43 (98%) | 0/6 (0%) | 168/175 (96%) |
| Stillbirth | 0/74 (0%) | 0/52 (0%) | 1/43 (2.3%) | 0/6 (0%) | 1/175 (0.6%) |
| Loss Before 24 Weeks | 0/74 (0%) | 0/52 (0%) | 0/43 (0%) | 6/6 (100%) | 6/175 (3.4%) |
| Gestation at Delivery (Weeks) |  |  |  |  |  |
| Mean (SD) | 37.6 (0.4) | 35.2 (1.2) | 32.5 (4.0) | 17.4 (3.2) | 35.0 (4.5) |
| Median (Q1, Q3) | 37.7 (37.1, 38.0) | 35.1 (34.7, 36.3) | 32.9 (29.9, 36.1) | 16.9 (14.0, 21.1) | 36.6 (34.7, 37.4) |
| Min, Max | 37.0, 38.4 | 31.9, 36.9 | 23.1, 36.9 | 14.0, 21.3 | 14.0, 38.4 |
|  |  |  |  |  |  |
| --- | --- | --- | --- | --- | --- |
| 1n/N (%) | | | | | |

```
multiples_neonatal_outcomes
```

| [B] Neonatal Outcomes - Multiples | | | | |
| --- | --- | --- | --- | --- |
|  | Term, N = 74 (44%)1 | iPTB, N = 52 (31%)1 | sPTB, N = 42 (25%)1 | Overall, N = 168 (100%)1 |
| Admission to SCBU or NICU | 2/74 (2.7%) | 20/50 (40%) | 26/42 (62%) | 48/166 (29%) |
| Not Reported | 0/74 (0%) | 2/52 (3.8%) | 0/42 (0%) | 2/168 (1.2%) |
| Number of inpatient nights |  |  |  |  |
| Mean (SD) | 3.6 (2.1) | 9.6 (9.5) | 31.5 (33.4) | 12.5 (20.9) |
| Median (Q1, Q3) | 4.0 (2.0, 6.0) | 6.0 (4.0, 11.0) | 15.5 (7.0, 52.0) | 5.0 (3.0, 10.0) |
| Min, Max | 0.0, 8.0 | 1.0, 50.0 | 3.0, 159.0 | 0.0, 159.0 |
| Not Reported | 0/74 (0%) | 2/52 (3.8%) | 0/42 (0%) | 2/168 (1.2%) |
|  |  |  |  |  |
| --- | --- | --- | --- | --- |
| 1n/N (%) | | | | |

Note**Supplementary Table F**: Pregnancy and delivery outcomes among multiple pregnancies in the INSIGHT cohort

Outcomes are summarised for participants with multiple pregnancies, summarised on a per-baby basis. [A] presents pregnancy outcomes and [B] presents neonatal outcomes. All multiple pregnancies in the cohort were twins. **Important**: One twin pregnancy contributes an odd number of outcome records because one twin was classified as a missed miscarriage before 13 weeks; no further outcome data were available for this baby because gestation at loss and other delivery/outcome details were unknown. The co-twin was later born following spontaneous preterm birth.

## 5. Sample Collection

```
#Add Formatted Weeks
Specimens.DF$`Gestation at Visit (Weeks)` <- (7*Specimens.DF$`Gestation at visit (w)`+Specimens.DF$`Gestation at visit (d)`)/7

# Dataframe has one row per aliquot, count aliquots by distinct specimen (Visit /Type)
Samples <- Specimens.DF %>% count(`Participant ID`,`Gestation at Visit (Weeks)`, `Specimen Type`)

# Echo total number samples/specimens and participant sampling visits
Total_Specimens <- nrow(Samples)
Total_Specimens
```

```
[1] 34863
```

```
Total_Participant_Sample_Visits <- Samples %>% select(`Participant ID`,`Gestation at Visit (Weeks)`) %>% distinct() %>% nrow()
Total_Participant_Sample_Visits
```

```
[1] 4999
```

```
#Retrieve Outcomes
Tmp <- Outcome.DF %>% select(`Participant ID`,PTB_Type_Formatted) %>% distinct()

#Check if twins result in two different outcomes for a participant (it does not)
Check <- Tmp %>% count(`Participant ID`)
max(Check$n)
```

```
[1] 1
```

```
#Merge in Outcomes
Samples <- merge(Samples,Tmp,by=c("Participant ID"),all.x = TRUE)

#Classify Gestation Period at Sample Collection
Samples$`Gestation at Sampling` <- Samples %>% mutate(
  LS = case_when(
    `Gestation at Visit (Weeks)` >= 20                          ~ "20+0-24+6 Weeks",
    `Gestation at Visit (Weeks)` >= 16                          ~ "16+0-19+6 Weeks",
    `Gestation at Visit (Weeks)` >= 14                          ~ "14+0-15+6 Weeks",
    `Gestation at Visit (Weeks)` >= 8                           ~ "8+0-13+6 Weeks",
    TRUE                                                        ~ "NA")) %>%
  pull(LS)

#Exclude Sample Types belonging to Other Studies
Samples <- Samples[grep("Neonatal|Child|Hair|Placenta|PBMC|Ficoll|HVS$",Samples$`Specimen Type`,ignore.case = TRUE,invert = TRUE),]
Samples <- Samples[Samples$`Gestation at Visit (Weeks)` <25,]

#Set Levels for Visualization
Samples$`Gestation at Sampling` <- factor(Samples$`Gestation at Sampling`,
                                          levels = c("20+0-24+6 Weeks","16+0-19+6 Weeks","14+0-15+6 Weeks","8+0-13+6 Weeks"))

#Formatting text 
Samples$`Specimen Type` <- str_replace(Samples$`Specimen Type`,"INSIGHT:","")
Samples[Samples$`Specimen Type` %in% "High vaginal swab (CVF)","Specimen Type"] <- "High vaginal fluid"
Samples[Samples$`Specimen Type` %in% "Low vaginal swab","Specimen Type"] <- "Low vaginal fluid"
Samples[Samples$`Specimen Type` %in% "Cell pellets (HVS)","Specimen Type"] <- "High vaginal cell pellet"
Samples[Samples$`Specimen Type` %in% "Cell pellets (CBS)","Specimen Type"] <- "Cytobrush cell pellet"
Samples[Samples$`Specimen Type` %in% "Microbiology swab 1-Slide (NS)","Specimen Type"] <- "Microbiology slide"
Samples[Samples$`Specimen Type` %in% "Microbiology swab 1-In Liquid Amies","Specimen Type"] <- "Swab stored in liquid Amies"
Samples[Samples$`Specimen Type` %in% "Microbiology swab 2-TE Buffer","Specimen Type"] <- "Swab stored in TE buffer"
Samples[Samples$`Specimen Type` %in% "Cytobrushing fluid","Specimen Type"] <- "Cytobrush fluid"

#Add Specimen Groups
Samples <- Samples %>%
  mutate(`Specimen Group` = case_when(
    `Specimen Type` %in% c("Plasma", "Serum", "Buffy coat") ~ "Venous Blood",
    `Specimen Type` %in% c("Cytobrush fluid", "Cytobrush cell pellet") ~ "Cytobrush",
    `Specimen Type` %in% c("High vaginal fluid", "High vaginal cell pellet") ~ "High Vaginal Swab",
    `Specimen Type` %in% c("Microbiology slide", 
                           "Swab stored in liquid Amies", "Swab stored in TE buffer") ~ "Microbiology Swab",
    `Specimen Type` %in% c("Low vaginal fluid", "Low vaginal cell pellet") ~ "Low Vaginal Swab",
    TRUE ~ NA
  ))

#Compile Aliquot Details
Aliquot_Details.df <- Samples %>% 
  group_by(`Specimen Type`,`Specimen Group`) %>%
  summarise(`Mean Aliquots` = round(mean(n),digits=2),
            `Median Aliquots` = median(n),
            .groups = "drop")

#Simplify to one row per participant for each gestational window and specimen type
Samples.Participants <- Samples %>% 
  count(`Participant ID`,`Specimen Type`,`Specimen Group`,`Gestation at Sampling`,PTB_Type_Formatted)

#Check how many times participants gave a specimen of a given type more than once in a gestational window (very uncommon)
Samples.Participants  %>% count(n)
```

```
Storing counts in `nn`, as `n` already present in input
ℹ Use `name = "new_name"` to pick a new name.
```

```
  n    nn
1 1 33935
2 2   343
3 3    14
```

```
#Total number of participants in a gestational window who gave at least one specimen of a given type
Samples <- Samples.Participants %>% count(`Specimen Type`,`Specimen Group`,`Gestation at Sampling`,PTB_Type_Formatted)

#Create Dataframe for Plotting
Plot.df <- Samples 

#Create Dataframe for Displaying
Sample_Details.df <- Samples %>% rename(`Pregnancy Outcome` = PTB_Type_Formatted) %>%
                                  dcast(`Specimen Type`+`Pregnancy Outcome`+`Specimen Group` ~ `Gestation at Sampling`,
                                        value.var = "n",fill = 0)

#Compile Visit Completeness Details (Visits where participants gave samples) and Upset Plot
 Visit_Details.df <- Samples.Participants %>% 
                    select(`Participant ID`,`Gestation at Sampling`) %>% 
                    distinct() %>% 
                    count(`Participant ID`,`Gestation at Sampling`) %>%
                    dcast(`Participant ID`~`Gestation at Sampling`,fill = 0)
```

```
Using n as value column: use value.var to override.
```

```
UpsetPlot <- UpSetR::upset(Visit_Details.df[,-1], text.scale = c(1.5, 1.5, 1.3, 1.3, 1.4, 1.1))
jpeg("../Stored_Resources/Figures/Plot.UPSETVisits.jpeg", width = 9, height = 5, units = "in", res = 300)
UpsetPlot
dev.off()
```

```
quartz_off_screen 
                2
```

```
Visit_Details.df <- Visit_Details.df %>% count(`20+0-24+6 Weeks`,`16+0-19+6 Weeks`,`14+0-15+6 Weeks`,`8+0-13+6 Weeks`)

#Omit the Single Miscarriage Blood Samples from the Plot for Better Visualization (this is mentioned in the legend)
Plot.df <- Plot.df %>% filter(!PTB_Type_Formatted %in% "Miscarriage")
SampleCounts.Plot <- ggplot(Plot.df,aes(y=`Specimen Type`,x=n,fill=`Gestation at Sampling`))+
                            geom_col(color="black")+
                            scale_fill_manual(values = c("#b51963","#8babf1","#f57600","#054fb9"))+
                            theme_bw()+
                            facet_grid(`Specimen Group`~PTB_Type_Formatted,scales="free")+
                            xlab("Number of Participants Per Gestational Window")

jpeg("../Stored_Resources/Figures/Plot.SampleCounts.jpeg", width = 10, height = 7, units = "in", res = 300)
SampleCounts.Plot
dev.off()
```

```
quartz_off_screen 
                2
```

```
#Compile Statistics on Participants who did not end up contributing Biological Specimens ###########

StudyParticipant.ID <- unique(Intake.DF$`Participant ID`)
SampleDonaters.ID <- unique(Specimens.DF$`Participant ID`)
ParticipantNoSample.ID <- setdiff(StudyParticipant.ID,SampleDonaters.ID)

ParticipantNoSample.Centre <- Intake.DF %>% select(`Participant ID`,Centre) %>%
                               filter(`Participant ID` %in% ParticipantNoSample.ID)

ParticipantNoSample.PrimaryOutcome <- Outcome.DF %>% 
  select(`Participant ID`,PTB_Type_Formatted) %>%
  filter(`Participant ID` %in% ParticipantNoSample.ID)  %>%
  distinct()

Tmp <- merge(ParticipantNoSample.Centre,ParticipantNoSample.PrimaryOutcome,by="Participant ID",
             all = TRUE)

#Check if any double outcomes (there are none) 
length(ParticipantNoSample.ID) == nrow(Tmp)
```

```
[1] TRUE
```

```
ParticipantNoSample.Details <- Tmp %>% count(Centre,PTB_Type_Formatted) %>% 
                               dcast(PTB_Type_Formatted~Centre,value.var = "n",
                                     fill=0) %>%
                              rename(`Pregnancy Outcome` = PTB_Type_Formatted)

ParticipantNoSample.Details <- ParticipantNoSample.Details %>% 
  flextable() %>%
  flextable::line_spacing(space = 1.1, part = "body") %>% 
  fontsize(size = 9, part = "all") %>%
  padding(padding = 2, part = "all") %>%
  line_spacing(space = 0.9, part = "all") %>%
  width(j = 1, width = 2.0) %>%
  width(j = 2, width = 4.0) %>%
  set_table_properties(
    layout = "fixed",
    align = "center"
  )
```

### **Figure 4**: Specimen Collection

```
SampleCounts.Plot
```

Note**Figure 4**

Distribution of participants contributing each specimen type, stratified by primary pregnancy outcome as defined in the Pregnancy and Delivery Outcomes section. Within each outcome stratum, each coloured segment represents the number of distinct participants who contributed at least one specimen of the specified type during the relevant gestational sampling window. A participant who contributed multiple specimens of the same type within a given window is counted only once in that segment. However, participants may be counted in multiple segments if they contributed specimens during more than one gestational window. Consequently, the total length of a stacked bar represents the sum of the window-specific participant counts and does not necessarily equal the number of unique participants who contributed that specimen type. Facet scales are allowed to vary for readability. One participant who experienced miscarriage and contributed three specimens at their first and only visit was omitted from the plot for readability; complete sample collection counts, including data from this participant, are provided in Supplementary Table G.

### **Supplementary Table G**: Sample Collection Counts

```
DT::datatable(
  Sample_Details.df, extensions = "Buttons", rownames = FALSE,
  options = list(
    dom = "Bfrtip",
    buttons = list(list(extend = "copy",text = "Copy table")),
    pageLength = 10))
```

Note**Supplementary Table G**

Counts of participants contributing each specimen type, stratified by pregnancy outcome and gestational sampling window.

### **Supplementary Table H**: Aliquot Collection Statistics

```
DT::datatable(
  Aliquot_Details.df, extensions = "Buttons", rownames = FALSE,
  options = list(
    dom = "Bfrtip",
    buttons = list(list(extend = "copy",text = "Copy table")),
    pageLength = 10))
```

Note**Supplementary Table H**

Mean and median number of aliquots obtained from an individual sample collection, summarised by specimen type and specimen group. For example, this reflects the typical number of aliquots generated when a given specimen type, such as plasma, was collected from a participant.

### **Figure 5**: Participant Visit Pattern (UpSet Plot)

```
UpsetPlot
```

Note**Figure 5**

UpSet plot showing patterns of biological specimen contribution across gestational sampling windows in the INSIGHT cohort. Each set represents a gestational window in which participants contributed at least one biological specimen. Set size indicates the total number of participants who contributed a specimen within each window, irrespective of attendance at other windows. Intersection size indicates the number of participants who contributed specimens in each specific combination of gestational windows. Solid black circles indicate included sampling windows, and connecting black lines indicate participants who contributed specimens across more than one window.

### **Supplementary Table I**: Participant Visit Pattern (Table)

```
DT::datatable(
  Visit_Details.df, extensions = "Buttons", rownames = FALSE,
  options = list(
    dom = "Bfrtip",
    buttons = list(list(extend = "copy",text = "Copy table")),
    pageLength = 10))
```

Note**Supplementary Table I**

Participant attendance patterns across gestational sampling windows. Each row represents a distinct combination of completed sampling windows, with counts indicating the number of participants who contributed at least one biological specimen in those windows. Data is visualised in Figure 5.  
  
High-vaginal cell pellets were retained only from February 2015 onward, and low-vaginal specimens were introduced in May 2016. Consequently, availability/missingness for these sample types is partly structural and associated with participants’ year of recruitment.

### **Supplementary Table J**: Participants Without Samples

```
ParticipantNoSample.Details
```

| Pregnancy Outcome | GSTT | MAN | PMH |
| --- | --- | --- | --- |
| Term | 45 | 4 | 6 |
| iPTB | 4 | 0 | 0 |
| sPTB | 14 | 0 | 0 |
| Mid-trimester Loss | 2 | 0 | 0 |

Note**Supplementary Table J**: Participants Without Samples

Pregnancy outcomes and recruitment sites for participants who were enrolled in the study but did not contribute biological specimens.

# Software and Versions

```
lock <- jsonlite::read_json("../renv.lock")

versions <- data.frame(
  Software = c("R", names(lock$Packages)),
  Version  = c(
    lock$R$Version,
    vapply(lock$Packages, `[[`, character(1), "Version")
  )
)

DT::datatable(
  versions, extensions = "Buttons", rownames = FALSE,
  options = list(
    dom = "Bfrtip",
    buttons = list(list(extend = "copy",text = "Copy table")),
    pageLength = 10))
```


##### Source Code

```
---
title: "Investigation into Biomarkers to Predict Preterm Birth (INSIGHT): a longitudinal UK pregnancy cohort for spontaneous preterm birth research - Publication Script"
author: "Raphaella Jackson"
date-modified: now
format:
  html:
    grid:
      sidebar-width: 250px
      body-width: 1000px
      margin-width: 50px
      gutter-width: 1.5rem
editor: visual
toc: true
toc-expand: true
toc-depth: 6
number-sections: false
toc-location: left
embed-resources: true
code-fold: false
code-tools: true
code-copy: false
code-block-bg: true
code-block-border-left: "#31BAE9"
---

# Guide

This document presents the code used to produce the statistics, figures, tables, and supplementary material included in the paper. The table of contents on the left can be used to navigate between outputs:    \
    \
- [figures are highlighted in **red**]{style="color: red;"}    \
- [tables in **purple**]{style="color: purple;"}    \
- [supplementary tables in **orange**]{style="color: orange;"}  \
- [supplementary figures in **blue**]{style="color: blue;"}  \

To view the underlying source code, click the Code button in the top-right corner of the document.

# Setup

## Load Packages

```{r,load_packages}

#Load Packages
library(readxl)
library(tidyverse)
library(consort)
library(flextable)
library(gtsummary)
library(UpSetR)
library(reshape2)

# Packages used but not fully loaded
# library(dlookR)
# library(sf)

```

## Load Data 

```{r,load_data}

#Clear Data Space (Sanity Clear)
rm(list=ls())

#Load Data
Intake.DF <- read.csv(file = "../Data/Intake.Dataframe.csv",check.names = FALSE)
Outcome.DF <- read.csv(file = "../Data/Outcomes.Dataframe.csv",check.names = FALSE)
Visits.DF <- read.csv(file = "../Data/Visits.Dataframe.csv",check.names = FALSE)
Specimens.DF <- read.csv(file = "../Data/Specimens.Dataframe.csv",check.names = FALSE)
ParticipantCounts.DF <- read.csv(file="../Data/ParticipantCounts.csv",check.names = FALSE)
SpecimenDetails.DF <- read.csv(file="../Data/Specimen_Details.csv",check.names = FALSE)
LTF.DF <- read.csv(file="../Data/LTF.Dataframe.csv",check.names = FALSE)

IMD.Info <- read.csv(file = "../Data/IMDInformation.csv",check.names = FALSE)

```

# **Methods**

## 1. Setting

### First and Last Visit Recorded

```{r}
First_Visit <- min(as.Date(Visits.DF$`Date of visit`,format="%Y-%m-%d"))
Last_Visit <- max(as.Date(Visits.DF$`Date of visit`,format="%Y-%m-%d"))
```

::: {.callout-note appearance="minimal" title="Quick Facts"}
The first visit of the study was conducted `r toString(First_Visit)` .\
The last visit of the study was conducted `r toString(Last_Visit)` .
:::

## 2. Recruitment and Eligibility

```{r,Flow_Code}

#Enrolled/Consented
Total_Enrolled.Count <- sum(colSums(ParticipantCounts.DF[,-1]))

#Exclusions
Lost_to_Follow_Up_Late.Count <- sum(ParticipantCounts.DF$Lost_to_Follow_Up_Late)
Lost_to_Followup_Early.Count <- sum(ParticipantCounts.DF$Lost_to_Follow_Up_Early)
Terminations.Count <- sum(ParticipantCounts.DF$Terminations)

All_Excluded.Count <- Lost_to_Follow_Up_Late.Count + Lost_to_Followup_Early.Count + Terminations.Count

#Retained for Analysis
Analysed.Count <- nrow(Intake.DF)

#Site Counts
GSTT.Count <- Intake.DF %>% filter(Centre %in% "STH") %>% nrow()
PMH.Count <- Intake.DF %>% filter(Centre %in% "PMH") %>% nrow()
MAN.Count <- Intake.DF %>% filter(Centre %in% "MAN") %>% nrow()
WM.Count <- Intake.DF %>% filter(Centre %in% "WM") %>% nrow()

### Flow Plot ###############################################################

#Generate Text
Consented_Box <- paste("Consented (n = ",Total_Enrolled.Count,")",sep = "")
Exclude_Aside <- paste("Excluded (",
                       All_Excluded.Count,
                       "):\n• Lost to Follow-up before Delivery (n = ",
                       Lost_to_Followup_Early.Count,
                       ")\n• Lost to Follow-up at Delivery (n = ",
                       Lost_to_Follow_Up_Late.Count,
                       ")\n• Terminations (n = ",
                       Terminations.Count, 
                       ")\n• Withdrew (n = 0)",
                       sep = "")
Analysed_Box <- paste("Retained for Analysis (n = ",Analysed.Count,")",sep = "")
Arm.GSTT <- paste( "GSTT (n = ",GSTT.Count,")",sep="")
Arm.PMH <- paste( "PMH  (n = ",PMH.Count,")",sep="")
Arm.MAN <- paste( "MAN (n = ",MAN.Count,")",sep="")
Arm.WM <- paste( "WM (n = ",WM.Count,")",sep="")

g <- add_box(,txt = Consented_Box)
g <- add_side_box(g,txt = Exclude_Aside)
g <- add_box(g,txt = Analysed_Box)
g <- add_split(g, txt = c(Arm.GSTT, Arm.PMH,Arm.MAN,Arm.WM))

jpeg("../Stored_Resources/Figures/Plot.Consort.jpeg", width = 10, height = 5, units = "in", res = 300)
g
dev.off()

flow_plot <- g

### Site Specific Table ###############################################################

ParticipantCounts.DF$Consented <- rowSums(ParticipantCounts.DF[,-1])

Out_Table <- ParticipantCounts.DF %>% select(Centre,Consented, Lost_to_Follow_Up_Early,Terminations,Lost_to_Follow_Up_Late,Enrolled) %>% 
    rename(`Lost to Follow-up - Before Delivery` = Lost_to_Follow_Up_Early,
           `Lost to Follow-up - At Delivery` = Lost_to_Follow_Up_Late,
           `Total Retained for Analysis` = Enrolled,
           `Total Consented` = Consented)

Out_Table[5,] <- append("All Sites",colSums(Out_Table[,-1]))

Site_Table <- Out_Table %>% 
  flextable() %>% 
  flextable::vline(j=1) %>% 
  flextable::hline(i=4) %>%
  flextable::line_spacing(space = 1.1, part = "body") %>% 
  flextable::autofit()

```
### [**Figure 1**]{style="color: red;"}: Participant Flow Graph

```{r,Flow_Output}
#| fig-width: 10
#| fig-height: 5

flow_plot

```

::: {.callout-note appearance="note" title="**Figure 1**: Participant Flow Diagram"}
Flow of participants from consent to inclusion in the analysis cohort, with retained participants stratified by recruitment site. “Lost to follow-up before delivery” refers to participants who consented but did not complete any research visits. “Lost to follow-up at delivery” refers to participants for whom basic delivery outcome data, including gestation at delivery and mode of delivery, could not be collected. GSTT denotes Guy’s and St Thomas’ NHS Foundation Trust, London; MAN denotes St Mary’s Hospital, Manchester University NHS Foundation Trust; PMH denotes St Mary’s Hospital, University Hospitals Dorset NHS Foundation Trust; WM denotes West Middlesex University Hospital, Chelsea and Westminster Hospital NHS Foundation Trust. A site-specific breakdown of consented, excluded, and retained participants is provided in Supplementary Table A.
:::

### [**Supplementary Table A**]{style="color: orange;"}:  Site Specific Participant Counts

```{r}
Site_Table
```

::: {.callout-note appearance="note" title="**Supplementary Table A**: Site Specific Participant Counts"}
Participant recruitment, exclusion, and retention counts by study site, with totals across all sites.
:::

## 4. Data Collection and Clinical Variables

```{r}
### Intake #####################################################################

#Diagnose
Intake.Diagnosis <- dlookr::diagnose(Intake.DF)
Intake.Diagnosis.Publish <- Intake.Diagnosis %>% select(variables,missing_count,missing_percent)

#Give Better Names
Intake.Diagnosis.Publish[Intake.Diagnosis.Publish$variables %in% "Centre","variables"] <- "Site"
Intake.Diagnosis.Publish[Intake.Diagnosis.Publish$variables %in% "Final agreed EDD","variables"] <- "Estimated Delivery Date"

#Add Type
Intake.Diagnosis.Publish$Variable_Type <- rep("Intake",nrow(Intake.Diagnosis.Publish))

### Outcomes ###################################################################

#Diagnose
Outcomes.Diagnosis <- dlookr::diagnose(Outcome.DF)
Outcomes.Diagnosis.Publish <- Outcomes.Diagnosis %>% select(variables,missing_count,missing_percent)

#Give Better Names
Outcomes.Diagnosis.Publish$variables <- str_replace(Outcomes.Diagnosis.Publish$variables, "Did she receive","Received")
Outcomes.Diagnosis.Publish$variables <- str_remove(Outcomes.Diagnosis.Publish$variables, "\\?")
Outcomes.Diagnosis.Publish[Outcomes.Diagnosis.Publish$variables %in% "Mag Sulph for fetal neuroprotection","variables"] <- "Received magnesium sulphate for fetal neuroprotection"
Outcomes.Diagnosis.Publish[Outcomes.Diagnosis.Publish$variables %in% "CRP","variables"] <- "Elevated CRP"
Outcomes.Diagnosis.Publish[Outcomes.Diagnosis.Publish$variables %in% "WCC","variables"] <- "Elevated WCC"

Outcomes.Diagnosis.Publish$Variable_Type <- rep("Outcome",nrow(Outcomes.Diagnosis.Publish))

### Combine ####################################################################

VariableSummary <- bind_rows(Intake.Diagnosis.Publish,Outcomes.Diagnosis.Publish)
VariableSummary$missing_percent <- round(VariableSummary$missing_percent,digits=2)
VariableSummary <- VariableSummary %>% rename(`Percent Unreported` = missing_percent,
                                              `Count Unreported` = missing_count,
                                              `Variable Type` = Variable_Type,
                                              `Variable` = variables)
write.csv(VariableSummary,file="../Stored_Resources/Tables/Supplementary.Table.VariableSummary.csv",row.names = FALSE)

```

### [**Supplementary Table B**]{style="color: orange;"}:  All Recorded Variables and Completeness

```{r}
DT::datatable(
  VariableSummary, extensions = "Buttons", rownames = FALSE,
  options = list(
    dom = "Bfrtip",
    buttons = list(list(extend = "copy",text = "Copy table")),
    pageLength = 10))

```

::: {.callout-note appearance="note" title="**Supplementary Table B**: Dataset Completeness"}
Completeness of recorded intake and outcome variables in the study dataset. **Please Note**:Intake variables are recorded once per participant; outcome variables are recorded once per baby.
:::

## 5. Biological Sample Collection and Processing
```{r}
### Specimen Details to Visualize
Specimen_Details_Table <-
  SpecimenDetails.DF %>%
  flextable() %>%
  set_header_labels(
    values = setNames(
      tools::toTitleCase(gsub("_", " ", names(SpecimenDetails.DF))),
      names(SpecimenDetails.DF)
    )
  ) %>%
  theme_vanilla() %>%
  bg(part = "header", bg = "#2F5597") %>%
  color(part = "header", color = "white") %>%
  bold(part = "header") %>%
  align(part = "header", align = "center") %>%
  align_text_col(align = "left") %>%
  align_nottext_col(align = "right") %>%
  padding(padding = 5, part = "all") %>%
  valign(valign = "center", part = "all") %>%
  autofit() %>%
  set_table_properties(
    layout = "autofit",
    align = "center"
  )

### Save for inclusion into word document (manuscript)

Out_Details <- SpecimenDetails.DF %>%
  flextable() %>% 
  flextable::line_spacing(space = 1.1, part = "body") %>% 
  fontsize(size = 9, part = "all") %>%
  padding(padding = 2, part = "all") %>%
  line_spacing(space = 0.9, part = "all") %>%
  width(j = 1, width = 2.0) %>%
  width(j = 2, width = 4.0) %>%
  set_table_properties(
    layout = "fixed",
    align = "center"
  )

save_as_docx(Out_Details,path="../Stored_Resources/Tables/Table.SpecimenDetails.docx")

```

### [**Table 1**]{style="color: purple;"}: Specimen Source Summary
```{r}
Specimen_Details_Table
```

::: {.callout-note appearance="note" title="**Table 1**: Specimen Source Summary"}
Biological specimen types collected in the INSIGHT study, showing the sample source, the derived sample generated from that source, and the simplified sample name used throughout the manuscript and associated outputs. SST denotes serum separator tube; EDTA denotes ethylenediaminetetraacetic acid; TE denotes Tris-EDTA buffer. Microbiology swabs labelled “1” and “2” indicate two separate swabs collected using the same sampling procedure; the numbering distinguishes their downstream processing or storage purpose rather than a difference in collection method.
:::

# **Results**

## 2. Baseline Participant Characteristics

```{r,Demo_Summary_Code}

#Compute BMI
Intake.DF$BMI <- Intake.DF$Weight/((Intake.DF$Height/100)^2)

#Give Gestation at Recruitment in Weeks (and fractions thereof) for ease of interpretation
Intake.DF$`Gestation at Recruitment (Weeks)` <- Intake.DF$`Gestation at Recruitment`/7

#Add IMD Information
IMD_Select <- IMD.Info %>% 
                  select(`LSOA code (2011)`,
                         `Index of Multiple Deprivation (IMD) Decile (where 1 is most deprived 10% of LSOAs)`) %>%
                  rename(`Lower super output area` = "LSOA code (2011)")

Intake.DF <- merge(Intake.DF,IMD_Select,by="Lower super output area",all.x = TRUE)

#Correct Site Name for Display
Intake.DF[Intake.DF$Centre %in% "STH","Centre"] <- "GSTT"

gtsummary::theme_gtsummary_compact(set_theme = TRUE, font_size = 10)

adv <- Intake.DF %>% 
  select(`Age at Registration`,
         `Ethnic Background`,
         BMI,
         `Index of Multiple Deprivation (IMD) Decile (where 1 is most deprived 10% of LSOAs)`,
         `Gestation at Recruitment (Weeks)`,
         Primigravida,
         `Pregnant with multiples`
         ) %>%
  tbl_summary(
    percent="column",
    type = all_continuous() ~ "continuous2",
    statistic = list(
      all_continuous() ~ c(
        "{mean} ({sd})",
        "{median} ({p25}, {p75})",
        "{min}, {max}"
      ),
      all_categorical() ~ "{n} ({p}%)"
    ),
    digits = list(all_continuous() ~ 1),
    missing = "ifany",
    missing_text = "Not Reported",
    missing_stat = "{N_miss} ({p_miss}%)"
  ) %>%
  bold_labels() %>%
  gtsummary::as_flex_table()

demographics_table <- adv %>% 
  flextable::line_spacing(space = 1.1, part = "body") %>% 
  fontsize(size = 9, part = "all") %>%
  padding(padding = 2, part = "all") %>%
  line_spacing(space = 0.9, part = "all") %>%
  width(j = 1, width = 2.0) %>%
  width(j = 2, width = 4.0) %>%
  set_table_properties(
    layout = "fixed",
    align = "center"
  )

save_as_docx(demographics_table,path="../Stored_Resources/Tables/Table.DemographicRisk.docx")

############# Comparative Demographics: By Site #############################################

gtsummary::theme_gtsummary_compact(set_theme = TRUE, font_size = 10)

adv <- Intake.DF %>% 
  select(Centre,
         `Age at Registration`,
         `Ethnic Background`,
         BMI,
         `Index of Multiple Deprivation (IMD) Decile (where 1 is most deprived 10% of LSOAs)`,
         `Gestation at Recruitment (Weeks)`,
         Primigravida,
         `Pregnant with multiples`,
         `High Risk`
         ) %>%
  tbl_summary(
    by = Centre,
    percent="column",
    type = all_continuous() ~ "continuous2",
    statistic = list(
      all_continuous() ~ c(
        "{mean} ({sd})",
        "{median} ({p25}, {p75})",
        "{min}, {max}"
      ),
      all_categorical() ~ "{n}/{N} ({p}%)"
    ),
    digits = list(all_continuous() ~ 1),
    missing = "ifany",
    missing_text = "Not Reported",
    missing_stat = "{N_miss} ({p_miss}%)"
  ) %>%
  bold_labels() %>%
  gtsummary::as_flex_table()

demographics_table_sitewise <- adv %>% 
  flextable::line_spacing(space = 1.1, part = "body") %>% 
  flextable::add_header_lines(values = "[A] Demographic Comparison - Sitewise") %>%
  flextable::bold(i = 1, bold = TRUE, part = "header") %>%
  flextable::align(i = 1, align = "center", part = "header") %>%
  fontsize(size = 9, part = "all") %>%
  padding(padding = 2, part = "all") %>%
  line_spacing(space = 0.9, part = "all") %>%
  width(j = 1, width = 2.0) %>%
  width(j = 2, width = 4.0) %>%
  set_table_properties(
    layout = "fixed",
    align = "center"
  )

############# Comparative Demographics: Lost to Follow-Up ##################

#Add IMD Information to LTF Dataset
IMD_Select <- IMD.Info %>% 
                  select(`LSOA code (2011)`,
                         `Index of Multiple Deprivation (IMD) Decile (where 1 is most deprived 10% of LSOAs)`) %>%
                  rename(`Lower super output area` = "LSOA code (2011)")
LTF.DF <- merge(LTF.DF,IMD_Select,by="Lower super output area",all.x = TRUE)
LTF.DF$`Age at Registration` <- LTF.DF$`Age at registration`

#Combine LTF Dataset and Analysis Dataset
Full_Set <- bind_rows(LTF.DF,Intake.DF) %>% 
  select(`Age at Registration`,
         `Ethnic Background`,
         `Index of Multiple Deprivation (IMD) Decile (where 1 is most deprived 10% of LSOAs)`,
         `Gestation at Recruitment (Weeks)`,
         `High Risk`,
         LTF_Type) 

Full_Set[is.na(Full_Set$LTF_Type),"LTF_Type"] <- "Analysis Dataset"

LTF_Comparison <- Full_Set %>%
  tbl_summary(
    by= LTF_Type,
    percent="column",
    type = all_continuous() ~ "continuous2",
    statistic = list(
      all_continuous() ~ c(
        "{mean} ({sd})",
        "{median} ({p25}, {p75})",
        "{min}, {max}"
      ),
      all_categorical() ~ "{n}/{N} ({p}%)"
    ),
    digits = list(all_continuous() ~ 1),
    missing = "ifany",
    missing_text = "Not Reported",
    missing_stat = "{N_miss} ({p_miss}%)"
  ) %>%
  bold_labels() %>%
  gtsummary::as_flex_table()

LTF_Comparison <- LTF_Comparison %>% 
  flextable::line_spacing(space = 1.1, part = "body") %>% 
  flextable::add_header_lines(values = "[B] Demographic Comparison - Lost to Follow-Up") %>%
  flextable::bold(i = 1, bold = TRUE, part = "header") %>%
  flextable::align(i = 1, align = "center", part = "header") %>%
  fontsize(size = 9, part = "all") %>%
  padding(padding = 2, part = "all") %>%
  line_spacing(space = 0.9, part = "all") %>%
  width(j = 1, width = 2.0) %>%
  width(j = 2, width = 4.0) %>%
  set_table_properties(
    layout = "fixed",
    align = "center"
  )
```

```{r, Map_Code}
#| echo: false
#| fig-width: 20
#| fig-height: 20

# Boundary data source:
# https://borders.ukdataservice.ac.uk/bds.html

england_lsoa <- sf::st_read("../Data/LSOA_2011_Boundaries/england_lsoa_2011.shp",quiet=TRUE)

LSOA_Participants <- Intake.DF %>% pull(`Lower super output area`) %>% unique()
  
england_lsoa[england_lsoa$code %in% LSOA_Participants,"Type"] <- "Represented"
england_lsoa[is.na(england_lsoa$Type),"Type"] <- "Absent"

england_dissolved <- england_lsoa %>%
  dplyr::group_by(Type) %>%
  dplyr::summarise(do_union = TRUE, .groups = "drop")

Map <- ggplot() + 
  geom_sf(data = england_dissolved, linewidth = 0,linetype=0, aes(fill = Type), colour="grey")+
  theme_void()+
  scale_fill_manual(values = c("grey","navy"),name = "LSOA Status")+ theme(
  axis.title = element_blank(),
  axis.text  = element_blank(),
  axis.ticks = element_blank()
)+ theme(legend.position = "none")

jpeg("../Stored_Resources/Figures/Plot.Map.jpeg", width = 10, height = 8, units = "in", res = 72)
Map
dev.off()

```

### [**Table 2**]{style="color: purple;"}: Demographic Summary
```{r}
demographics_table
```

::: {.callout-note appearance="note" title="**Table 2**: Demographic Summary"}
Sociodemographic, anthropometric, pregnancy, and recruitment characteristics of participants included in the INSIGHT study cohort. Percentages are calculated using the full participant cohort (n=2272) as the denominator unless otherwise indicated. Ethnicity was self-reported and coded using categories informed by Office for National Statistics (ONS) classifications defined at study outset. For reporting, legacy category labels were updated to contemporary terminology: “AfroCaribbean” is reported as “Black Caribbean” and “Far East Asian” as “East Asian”; the underlying participant classifications were unchanged. Gestation at Recruitment reflects final dating following ultrasound. Where early ultrasound redating revised the estimated gestation, the recorded value at recruitment may fall below the 10+0 week enrolment threshold, accounting for the minimum of 8.0 weeks shown. 
:::

### [**Supplementary Table C [A and B]**]{style="color: orange;"}: Demographic Comparison: Sitewise and Lost to Follow-Up

```{r}
demographics_table_sitewise
LTF_Comparison
```

::: {.callout-note appearance="note" title="**Supplementary Table C**: Demographic Comparison: Lost to Follow-Up"}
A comparison of basic demographic characteristics of **[A]** participants retained within the analysis cohort broken down by site **[B]** all consented participants broken down by whether they were lost to follow-up early/late or retained in the analysis cohort. Ethnicity was self-reported and coded using categories informed by Office for National Statistics (ONS) classifications defined at study outset. For reporting, legacy category labels were updated to contemporary terminology: “AfroCaribbean” is reported as “Black Caribbean” and “Far East Asian” as “East Asian”; the underlying participant classifications were unchanged. High risk indicates the participant's final risk status determination based on the criteria listed under Recruitment and Eligibility, based on all the information available at the conclusion of the study. 
:::


### [**Figure 2**]{style="color: red;"}: Geographic Distribution
```{r}
#| fig-format: png
#| fig-width: 10
#| fig-height: 8
#| fig-dpi: 72
#| fig-retina: 1

Map
```

::: {.callout-note appearance="note" title="**Figure 2**:Map of Participant LSOAs"}
Map showing lower layer super output areas (LSOAs) represented by participants in the INSIGHT study cohort. LSOAs containing at least one participant are shaded navy; all other English LSOAs are shaded grey. LSOA denotes lower layer super output area, a small-area geographic unit used for reporting population statistics in England.
:::


## 3. Risk Stratification and Risk Factor Distribution


```{r,UpSet_Risk_Plot}

#The column for cervical length is stored as <25mm even though it should be ≤25mm because the ≤ symbol can't be stored in the database and isn't read in properly by R. As a result we fix the name after it is loaded.

High_Risk_Participants <- Intake.DF %>% filter(`High Risk` %in% "Yes")
High_Risk_Participants.Count <- nrow(High_Risk_Participants)

Cervix_Measured <- Intake.DF %>% filter(!`Cervical length <25mm (19-24+6 weeks)` %in% "Not Measured")
Cervix_Measured.Count <- nrow(Cervix_Measured)

HighRisk.Table <- High_Risk_Participants %>% select(`Participant ID`,
                                                    `Previous spontaneous PTB (<37 weeks)`,
                                                    `Previous midtrimester loss (16-23+6 weeks)`,
                                                    `Previous PPROM (<37 weeks)`,
                                                    `Cervical length <25mm (19-24+6 weeks)`,
                                                    `Previous cervical surgery (e.g. LLETZ, Cone)`,
                                                    `Pregnant with multiples`,
                                                    `Uterine abnormality`)


colnames(HighRisk.Table) <- c("Participant ID",
                              "Previous sPTB",
                              "Previous MTL",
                              "Previous PPROM",
                              "Cervical Length ≤ 25",
                              "Cervical History",
                              "Multiple Pregnancy",
                              "Uterine Variant")

Risk_Counts_All.df <- HighRisk.Table %>%
  count(across(all_of(colnames(HighRisk.Table[,-1]))), name = "Number of Participants")

HighRisk.Table$`*Cervix Not Measured` <- rep("No",nrow(HighRisk.Table))
HighRisk.Table[!HighRisk.Table$`Participant ID` %in% Cervix_Measured$`Participant ID`, "*Cervix Not Measured"] <- "Yes"

CombMat <- HighRisk.Table[,-1]
CombMat[CombMat=="Yes"] <- 1
CombMat[CombMat=="No"] <- 0
CombMat[CombMat=="Not Measured"] <- 0
CombMat <- CombMat %>% mutate_if(is.character, as.numeric)
CombMat <- CombMat[,colSums(CombMat)>0]
CombMat <- data.frame(CombMat,check.names = FALSE)

Risk_Counts_Individual.df <- CombMat %>%
  summarise(across(everything(), ~ sum(.x == 1, na.rm = TRUE))) %>%
  tidyr::pivot_longer(everything(),names_to = "Risk Factor",values_to = "Number of Participants")

UpsetPlot <- UpSetR::upset(CombMat,nsets = 25, text.scale = c(1.5, 1.5, 1.3, 1.3, 1.4, 1.1))

jpeg("../Stored_Resources/Figures/Plot.UPSETRisk.jpeg", width = 9, height = 5, units = "in", res = 300)
UpsetPlot
dev.off()
```

### Notes and Definitions
::: {.callout-note appearance="minimal" title="Risk Profiles of Participants"}
Participants with varying risk profiles for preterm birth were recruited.\
Here we consider participants to be at high risk of preterm birth if they fulfilled one or more of the following criteria which were specified as being high risk criteria for the INSIGHT study.

•   [Previous PTB] Previous preterm birth or mid-trimester loss between 16+0 and 36+6 weeks' gestation.    \
•   [Previous PPROM] Previous preterm prelabour rupture of the fetal membranes (PPROM) at ≤ 36+6 weeks' gestation.    \
•   [Cervical Length ≤25] Short cervical length (≤ 25 mm) on ultrasound between 18+0 and 24+0 weeks' gestation.    \
•   [Cervical History] Previous cervical procedure to treat abnormal smears (large loop excision, laser conisation, cold knife conisation, or radical diathermy).    \
•   [Multiple Pregnancy] Multiple pregnancy     \
•   [Uterine Variant] Known Müllerian Abnormalities    \

:::

::: {.callout-note appearance="minimal" title="Risk Profiles of Participants"}
A total of `r toString(High_Risk_Participants.Count)` high risk participants were recruited.
:::

### [**Figure 3**]{style="color: red;"}: Risk Factor UpSet Plot

::: {.callout-important appearance="important"}
Not having a measurement of the cervix is not considered a risk factor. However, we identify participants who were classified as high risk who did not have their cervix measured as it is not known whether they may have had a short cervix or not. 
:::

```{r}
#| fig-width: 10
#| fig-height: 6
UpsetPlot
```

::: {.callout-note appearance="note" title="**Figure 3**: Risk Factor UpSet Plot"}
UpSet plot showing the individual and overlapping risk factors used to classify participants as being at high risk of preterm birth. Set size indicates the number of participants with each risk factor irrespective of other co-occurring risk factors; intersection size indicates the number of participants with each specific combination of risk factors. Solid black circles indicate risk factors present in each group, and connecting black lines indicate combinations involving more than one risk factor. sPTB denotes spontaneous preterm birth; MTL denotes mid-trimester loss between 16+0 and 23+6 weeks' gestation (mid-trimester loss between 14+0 and 15+6 weeks’ gestation is not included in this criterion); PPROM denotes preterm prelabour rupture of membranes. Cervical history refers to previous cervical procedures. All risk factors are fully defined in the Recruitment and Eligibility section. The reported frequencies are the final, updated risk status of participants, in cases where participants had a cervical measurement ≤ 25 mm on ultrasound between 18+0 and 24+0 weeks' gestation which occurred after their enrolment visit.
:::

### [**Supplementary Table D**]{style="color: orange;"}: Risk Factor Table - All

```{r}
DT::datatable(
  Risk_Counts_All.df, extensions = "Buttons", rownames = FALSE,
  options = list(
    dom = "Bfrtip",
    buttons = list(list(extend = "copy",text = "Copy table")),
    pageLength = 10))

```

::: {.callout-note appearance="note" title="**Supplementary Table D**: Risk Factors (Intersecting)"}
Counts of distinct combinations of preterm birth risk factors among high-risk participants, corresponding to the intersection-size bars in Figure 3.
:::

### [**Supplementary Table E**]{style="color: orange;"}: Risk Factor Table - Individual

```{r}
DT::datatable(
  Risk_Counts_Individual.df, extensions = "Buttons", rownames = FALSE,
  options = list(
    dom = "Bfrtip",
    buttons = list(list(extend = "copy",text = "Copy table")),
    pageLength = 10))

```

::: {.callout-note appearance="note" title="**Supplementary Table E**: Risk Factors (Independent)"}
Counts of participants with each individual preterm birth risk factor, corresponding to the set-size bars in Figure 3.
:::

## 4. Pregnancy and Delivery Outcomes

```{r}
# Add Gestation at Delivery in Weeks plus Fraction of Weeks
Outcome.DF$`Gestation at Delivery (Weeks)` <- (Outcome.DF$`Gestation at delivery (w)`*7+Outcome.DF$`Gestation at delivery (d)`)/7

# Add Risk and Multiple Information to Table
T1 <- nrow(Outcome.DF)
Tmp <- Intake.DF %>% select(`Participant ID`,`High Risk`,`Pregnant with multiples`)
Outcome.DF <- merge(Outcome.DF,Tmp,by="Participant ID",all = TRUE)
T2 <- nrow(Outcome.DF)
stopifnot(T1 == T2)

#Make the formatting a bit nicer on the categories
Outcome.DF$PTB_Type_Formatted <- Outcome.DF %>% mutate(
  LS = case_when(
    PTB_Type %in% "Term"                       ~ "Term",
    PTB_Type %in% "IPTB"                       ~ "iPTB",
    PTB_Type %in% "SPTB"                       ~ "sPTB",
    PTB_Type %in% "MidTrimesterLoss_24"        ~ "Mid-trimester Loss",
    PTB_Type %in% "Miscarriage_14"             ~ "Miscarriage",
    TRUE                                       ~ "NA")) %>%
  pull(LS)

Outcome.DF$PTB_Type_Formatted <- factor(Outcome.DF$PTB_Type_Formatted ,
                                        levels = c("Term","iPTB","sPTB","Mid-trimester Loss","Miscarriage"))

#Group MTL and Miscarriage outcome to not be repetitive
Outcome.DF[Outcome.DF$PTB_Type_Formatted %in% c("Mid-trimester Loss","Miscarriage"),"Pregnancy Outcome"] <- "Loss Before 24 Weeks"
Outcome.DF$`Pregnancy Outcome` <- factor(Outcome.DF$`Pregnancy Outcome`, levels = c("Live birth","Stillbirth","Loss Before 24 Weeks"))

#Clarify the Stillbirth and Miscarriage are not applicable for NICU admission or inpatient nights
Outcome.DF <- Outcome.DF %>%
  mutate(Neonatal_Outcome_Applicable = !(PTB_Type_Formatted %in% c("Miscarriage","Mid-trimester Loss") |
                                         `Pregnancy Outcome` %in% "Stillbirth"),
    `Admission to SCBU or NICU` = if_else(Neonatal_Outcome_Applicable,
                                          `Admission to SCBU or NICU`,
      "Not Applicable"))

##### Singleton Outcomes #######################################################

# Singletons
Singles.Table_Values <- Outcome.DF %>%
  filter(`Pregnant with multiples` %in% "No") %>%
  mutate(
    Inpatient_Nights_Raw = as.character(`Number of inpatient nights`),
    NICU_Raw = as.character(`Admission to SCBU or NICU`),

    Neonatal_Applicable = !(
      Inpatient_Nights_Raw %in% "Not Applicable" |
      NICU_Raw %in% "Not Applicable"
    ),

    `Number of inpatient nights` = suppressWarnings(as.numeric(case_when(
      Inpatient_Nights_Raw %in% c("Not Applicable", "Not Reported", "NA", "") ~ NA_character_,
      TRUE ~ Inpatient_Nights_Raw
    ))),

    `Admission to SCBU or NICU` = case_when(
      NICU_Raw %in% c("Not Reported", "NA", "") ~ NA_character_,
      TRUE ~ NICU_Raw
    )
  )

#Outcomes - All Births
singleton_outcomes_tbl <- Singles.Table_Values %>%
  select(
    `High Risk`,
    `Pregnancy Outcome`,
    `Gestation at Delivery (Weeks)`,
    PTB_Type_Formatted
  ) %>%
  tbl_summary(
    by = PTB_Type_Formatted,
    percent = "column",
    type = all_continuous() ~ "continuous2",
    statistic = list(
      all_continuous() ~ c(
        "{mean} ({sd})",
        "{median} ({p25}, {p75})",
        "{min}, {max}"
      ),
      all_categorical() ~ "{n}/{N} ({p}%)"
    ),
    digits = list(all_continuous() ~ 1),
    missing = "ifany",
    missing_text = "Not Reported",
    missing_stat = "{N_miss}/{N_obs} ({p_miss}%)"
  ) %>%
  add_overall(last = TRUE) %>%
  bold_labels() %>%
  modify_header(
    label = "",
    all_stat_cols() ~ "**{level}**, N = {n} ({style_percent(p)}%)"
  ) %>%
  gtsummary::as_flex_table()

singletons_outcomes <- singleton_outcomes_tbl %>%
  flextable::vline(j = 1) %>%
  flextable::vline(j = 6) %>%
  flextable::add_header_lines(values = "[A] Pregnancy Outcomes - Singletons") %>%
  flextable::bold(i = 1, bold = TRUE, part = "header") %>%
  flextable::align(i = 1, align = "center", part = "header") %>%
  flextable::line_spacing(space = 1.1, part = "body") %>%
  flextable::autofit()

#Outcomes - Neonatal Care (Where applicable)

neonatal_outcomes_tbl <- Singles.Table_Values %>%
  filter(Neonatal_Applicable) %>%
  mutate(PTB_Type_Formatted = droplevels(PTB_Type_Formatted)) %>%
  select(
    `Admission to SCBU or NICU`,
    `Number of inpatient nights`,
    PTB_Type_Formatted
  ) %>%
  tbl_summary(
    by = PTB_Type_Formatted,
    percent = "column",
    type = all_continuous() ~ "continuous2",
    statistic = list(
      all_continuous() ~ c(
        "{mean} ({sd})",
        "{median} ({p25}, {p75})",
        "{min}, {max}"
      ),
      all_categorical() ~ "{n}/{N} ({p}%)"
    ),
    digits = list(all_continuous() ~ 1),
    missing = "ifany",
    missing_text = "Not Reported",
    missing_stat = "{N_miss}/{N_obs} ({p_miss}%)"
  ) %>%
  add_overall(last = TRUE) %>%
  bold_labels() %>%
  modify_header(
    label = "",
    all_stat_cols() ~ "**{level}**, N = {n} ({style_percent(p)}%)"
  ) %>%
  gtsummary::as_flex_table()

singletons_neonatal_outcomes <- neonatal_outcomes_tbl %>%
  flextable::vline(j = 1) %>%
  flextable::vline(j = 4) %>%
  flextable::add_header_lines(values = "[B] Neonatal Outcomes - Singletons") %>%
  flextable::bold(i = 1, bold = TRUE, part = "header") %>%
  flextable::align(i = 1, align = "center", part = "header") %>%
  flextable::line_spacing(space = 1.1, part = "body") %>%
  flextable::autofit()

save_as_docx(singletons_outcomes,path="../Stored_Resources/Tables/Table.Singletons.Outcomes.docx")
save_as_docx(singletons_neonatal_outcomes,path="../Stored_Resources/Tables/Table.Singletons.NeoOutcomes.docx")

##### Multiples Outcomes #######################################################

# Multplies
Multiples.Table_Values <- Outcome.DF %>%
  filter(`Pregnant with multiples` %in% "Yes") %>%
  mutate(
    Inpatient_Nights_Raw = as.character(`Number of inpatient nights`),
    NICU_Raw = as.character(`Admission to SCBU or NICU`),

    Neonatal_Applicable = !(
      Inpatient_Nights_Raw %in% "Not Applicable" |
      NICU_Raw %in% "Not Applicable"
    ),

    `Number of inpatient nights` = suppressWarnings(as.numeric(case_when(
      Inpatient_Nights_Raw %in% c("Not Applicable", "Not Reported", "NA", "") ~ NA_character_,
      TRUE ~ Inpatient_Nights_Raw
    ))),

    `Admission to SCBU or NICU` = case_when(
      NICU_Raw %in% c("Not Reported", "NA", "") ~ NA_character_,
      TRUE ~ NICU_Raw
    )
  )

#Outcomes - All Births
multiples_outcomes_tbl <- Multiples.Table_Values %>%
  select(
    `High Risk`,
    `Pregnancy Outcome`,
    `Gestation at Delivery (Weeks)`,
    PTB_Type_Formatted
  ) %>%
  mutate(PTB_Type_Formatted = droplevels(PTB_Type_Formatted)) %>%
  tbl_summary(
    by = PTB_Type_Formatted,
    percent = "column",
    type = all_continuous() ~ "continuous2",
    statistic = list(
      all_continuous() ~ c(
        "{mean} ({sd})",
        "{median} ({p25}, {p75})",
        "{min}, {max}"
      ),
      all_categorical() ~ "{n}/{N} ({p}%)"
    ),
    digits = list(all_continuous() ~ 1),
    missing = "ifany",
    missing_text = "Not Reported",
    missing_stat = "{N_miss}/{N_obs} ({p_miss}%)"
  ) %>%
  add_overall(last = TRUE) %>%
  bold_labels() %>%
  modify_header(
    label = "",
    all_stat_cols() ~ "**{level}**, N = {n} ({style_percent(p)}%)"
  ) %>%
  gtsummary::as_flex_table()

multiples_outcomes <- multiples_outcomes_tbl %>%
  flextable::vline(j = 1) %>%
  flextable::vline(j = 6) %>%
  flextable::add_header_lines(values = "[A] Pregnancy Outcomes - Multiples") %>%
  flextable::bold(i = 1, bold = TRUE, part = "header") %>%
  flextable::align(i = 1, align = "center", part = "header") %>%
  flextable::line_spacing(space = 1.1, part = "body") %>%
  flextable::autofit()

#Outcomes - Neonatal Care (Where applicable)
multiples_outcomes_tbl <- Multiples.Table_Values %>%
  filter(Neonatal_Applicable) %>%
  mutate(PTB_Type_Formatted = droplevels(PTB_Type_Formatted)) %>%
  select(
    `Admission to SCBU or NICU`,
    `Number of inpatient nights`,
    PTB_Type_Formatted
  ) %>%
  tbl_summary(
    by = PTB_Type_Formatted,
    percent = "column",
    type = all_continuous() ~ "continuous2",
    statistic = list(
      all_continuous() ~ c(
        "{mean} ({sd})",
        "{median} ({p25}, {p75})",
        "{min}, {max}"
      ),
      all_categorical() ~ "{n}/{N} ({p}%)"
    ),
    digits = list(all_continuous() ~ 1),
    missing = "ifany",
    missing_text = "Not Reported",
    missing_stat = "{N_miss}/{N_obs} ({p_miss}%)"
  ) %>%
  add_overall(last = TRUE) %>%
  bold_labels() %>%
  modify_header(
    label = "",
    all_stat_cols() ~ "**{level}**, N = {n} ({style_percent(p)}%)"
  ) %>%
  gtsummary::as_flex_table()

multiples_neonatal_outcomes <- multiples_outcomes_tbl %>%
  flextable::vline(j = 1) %>%
  flextable::vline(j = 4) %>%
  flextable::add_header_lines(values = "[B] Neonatal Outcomes - Multiples") %>%
  flextable::bold(i = 1, bold = TRUE, part = "header") %>%
  flextable::align(i = 1, align = "center", part = "header") %>%
  flextable::line_spacing(space = 1.1, part = "body") %>%
  flextable::autofit()

save_as_docx(multiples_outcomes,path="../Stored_Resources/Tables/Table.Multiples.Outcomes.docx")
save_as_docx(multiples_neonatal_outcomes,path="../Stored_Resources/Tables/Table.Multiples.NeoOutcomes.docx")

```

### [**Table 3**]{style="color: purple;"}: Singleton Outcomes

```{r}
singletons_outcomes
singletons_neonatal_outcomes

```

::: {.callout-note appearance="note" title="**Table 3**: Pregnancy and neonatal outcomes among participants with singleton pregnancies"}
Outcomes are summarised for participants with singleton pregnancies. Table 3A presents pregnancy outcomes, and Table 3B presents neonatal outcomes. Values are presented as n (%) unless otherwise stated. Percentages in Table 3A are calculated using the number of singleton pregnancies within each outcome group as the denominator; for singleton pregnancies, this is equivalent to the number of participants. Percentages in Table 3B are calculated using the number of singleton live births within each outcome group as the denominator; miscarriages, stillbirths, and mid-trimester losses are excluded from these denominators because admission to SCBU or NICU and inpatient neonatal stay are not applicable to these outcomes, while applicable outcomes not reported are retained as "Not Reported". Number of inpatient nights refers to the number of nights the infant remained admitted to hospital after birth. Term birth was defined as delivery at ≥37+0 weeks’ gestation. Spontaneous preterm birth (sPTB) was defined as delivery before 37+0 weeks’ gestation following spontaneous onset of labour or preterm prelabour rupture of membranes. Iatrogenic preterm birth (iPTB) was defined as delivery before 37+0 weeks’ gestation following medical intervention, including induction of labour or prelabour caesarean birth for maternal or fetal indications. Mid-trimester loss was defined as pregnancy loss between 14+0 and 23+6 weeks’ gestation. Stillbirth was defined as birth without signs of life at or beyond 24+0 weeks’ gestation. Miscarriage was defined as pregnancy loss before 14+0 weeks’ gestation with known outcome details; missed miscarriages are not included in this category. SCBU denotes Special Care Baby Unit; NICU denotes Neonatal Intensive Care Unit.
:::

### [**Supplementary Table F**]{style="color: orange;"}: Multiples Outcomes

```{r}
multiples_outcomes
multiples_neonatal_outcomes
```

::: {.callout-note appearance="note" title="**Supplementary Table F**: Pregnancy and delivery outcomes among multiple pregnancies in the INSIGHT cohort"}

Outcomes are summarised for participants with multiple pregnancies, summarised on a per-baby basis. [A] presents pregnancy outcomes and [B] presents neonatal outcomes. All multiple pregnancies in the cohort were twins. [**Important**]{style="color: red;"}: One twin pregnancy contributes an odd number of outcome records because one twin was classified as a missed miscarriage before 13 weeks; no further outcome data were available for this baby because gestation at loss and other delivery/outcome details were unknown. The co-twin was later born following spontaneous preterm birth.

:::

## 5. Sample Collection

```{r,Samples}

#Add Formatted Weeks
Specimens.DF$`Gestation at Visit (Weeks)` <- (7*Specimens.DF$`Gestation at visit (w)`+Specimens.DF$`Gestation at visit (d)`)/7

# Dataframe has one row per aliquot, count aliquots by distinct specimen (Visit /Type)
Samples <- Specimens.DF %>% count(`Participant ID`,`Gestation at Visit (Weeks)`, `Specimen Type`)

# Echo total number samples/specimens and participant sampling visits
Total_Specimens <- nrow(Samples)
Total_Specimens
Total_Participant_Sample_Visits <- Samples %>% select(`Participant ID`,`Gestation at Visit (Weeks)`) %>% distinct() %>% nrow()
Total_Participant_Sample_Visits

#Retrieve Outcomes
Tmp <- Outcome.DF %>% select(`Participant ID`,PTB_Type_Formatted) %>% distinct()

#Check if twins result in two different outcomes for a participant (it does not)
Check <- Tmp %>% count(`Participant ID`)
max(Check$n)

#Merge in Outcomes
Samples <- merge(Samples,Tmp,by=c("Participant ID"),all.x = TRUE)

#Classify Gestation Period at Sample Collection
Samples$`Gestation at Sampling` <- Samples %>% mutate(
  LS = case_when(
    `Gestation at Visit (Weeks)` >= 20                          ~ "20+0-24+6 Weeks",
    `Gestation at Visit (Weeks)` >= 16                          ~ "16+0-19+6 Weeks",
    `Gestation at Visit (Weeks)` >= 14                          ~ "14+0-15+6 Weeks",
    `Gestation at Visit (Weeks)` >= 8                           ~ "8+0-13+6 Weeks",
    TRUE                                                        ~ "NA")) %>%
  pull(LS)

#Exclude Sample Types belonging to Other Studies
Samples <- Samples[grep("Neonatal|Child|Hair|Placenta|PBMC|Ficoll|HVS$",Samples$`Specimen Type`,ignore.case = TRUE,invert = TRUE),]
Samples <- Samples[Samples$`Gestation at Visit (Weeks)` <25,]

#Set Levels for Visualization
Samples$`Gestation at Sampling` <- factor(Samples$`Gestation at Sampling`,
                                          levels = c("20+0-24+6 Weeks","16+0-19+6 Weeks","14+0-15+6 Weeks","8+0-13+6 Weeks"))

#Formatting text 
Samples$`Specimen Type` <- str_replace(Samples$`Specimen Type`,"INSIGHT:","")
Samples[Samples$`Specimen Type` %in% "High vaginal swab (CVF)","Specimen Type"] <- "High vaginal fluid"
Samples[Samples$`Specimen Type` %in% "Low vaginal swab","Specimen Type"] <- "Low vaginal fluid"
Samples[Samples$`Specimen Type` %in% "Cell pellets (HVS)","Specimen Type"] <- "High vaginal cell pellet"
Samples[Samples$`Specimen Type` %in% "Cell pellets (CBS)","Specimen Type"] <- "Cytobrush cell pellet"
Samples[Samples$`Specimen Type` %in% "Microbiology swab 1-Slide (NS)","Specimen Type"] <- "Microbiology slide"
Samples[Samples$`Specimen Type` %in% "Microbiology swab 1-In Liquid Amies","Specimen Type"] <- "Swab stored in liquid Amies"
Samples[Samples$`Specimen Type` %in% "Microbiology swab 2-TE Buffer","Specimen Type"] <- "Swab stored in TE buffer"
Samples[Samples$`Specimen Type` %in% "Cytobrushing fluid","Specimen Type"] <- "Cytobrush fluid"

#Add Specimen Groups
Samples <- Samples %>%
  mutate(`Specimen Group` = case_when(
    `Specimen Type` %in% c("Plasma", "Serum", "Buffy coat") ~ "Venous Blood",
    `Specimen Type` %in% c("Cytobrush fluid", "Cytobrush cell pellet") ~ "Cytobrush",
    `Specimen Type` %in% c("High vaginal fluid", "High vaginal cell pellet") ~ "High Vaginal Swab",
    `Specimen Type` %in% c("Microbiology slide", 
                           "Swab stored in liquid Amies", "Swab stored in TE buffer") ~ "Microbiology Swab",
    `Specimen Type` %in% c("Low vaginal fluid", "Low vaginal cell pellet") ~ "Low Vaginal Swab",
    TRUE ~ NA
  ))

#Compile Aliquot Details
Aliquot_Details.df <- Samples %>% 
  group_by(`Specimen Type`,`Specimen Group`) %>%
  summarise(`Mean Aliquots` = round(mean(n),digits=2),
            `Median Aliquots` = median(n),
            .groups = "drop")

#Simplify to one row per participant for each gestational window and specimen type
Samples.Participants <- Samples %>% 
  count(`Participant ID`,`Specimen Type`,`Specimen Group`,`Gestation at Sampling`,PTB_Type_Formatted)

#Check how many times participants gave a specimen of a given type more than once in a gestational window (very uncommon)
Samples.Participants  %>% count(n)

#Total number of participants in a gestational window who gave at least one specimen of a given type
Samples <- Samples.Participants %>% count(`Specimen Type`,`Specimen Group`,`Gestation at Sampling`,PTB_Type_Formatted)

#Create Dataframe for Plotting
Plot.df <- Samples 

#Create Dataframe for Displaying
Sample_Details.df <- Samples %>% rename(`Pregnancy Outcome` = PTB_Type_Formatted) %>%
                                  dcast(`Specimen Type`+`Pregnancy Outcome`+`Specimen Group` ~ `Gestation at Sampling`,
                                        value.var = "n",fill = 0)

#Compile Visit Completeness Details (Visits where participants gave samples) and Upset Plot
 Visit_Details.df <- Samples.Participants %>% 
                    select(`Participant ID`,`Gestation at Sampling`) %>% 
                    distinct() %>% 
                    count(`Participant ID`,`Gestation at Sampling`) %>%
                    dcast(`Participant ID`~`Gestation at Sampling`,fill = 0) 

UpsetPlot <- UpSetR::upset(Visit_Details.df[,-1], text.scale = c(1.5, 1.5, 1.3, 1.3, 1.4, 1.1))
jpeg("../Stored_Resources/Figures/Plot.UPSETVisits.jpeg", width = 9, height = 5, units = "in", res = 300)
UpsetPlot
dev.off()

Visit_Details.df <- Visit_Details.df %>% count(`20+0-24+6 Weeks`,`16+0-19+6 Weeks`,`14+0-15+6 Weeks`,`8+0-13+6 Weeks`)

#Omit the Single Miscarriage Blood Samples from the Plot for Better Visualization (this is mentioned in the legend)
Plot.df <- Plot.df %>% filter(!PTB_Type_Formatted %in% "Miscarriage")
SampleCounts.Plot <- ggplot(Plot.df,aes(y=`Specimen Type`,x=n,fill=`Gestation at Sampling`))+
                            geom_col(color="black")+
                            scale_fill_manual(values = c("#b51963","#8babf1","#f57600","#054fb9"))+
                            theme_bw()+
                            facet_grid(`Specimen Group`~PTB_Type_Formatted,scales="free")+
                            xlab("Number of Participants Per Gestational Window")

jpeg("../Stored_Resources/Figures/Plot.SampleCounts.jpeg", width = 10, height = 7, units = "in", res = 300)
SampleCounts.Plot
dev.off()

#Compile Statistics on Participants who did not end up contributing Biological Specimens ###########

StudyParticipant.ID <- unique(Intake.DF$`Participant ID`)
SampleDonaters.ID <- unique(Specimens.DF$`Participant ID`)
ParticipantNoSample.ID <- setdiff(StudyParticipant.ID,SampleDonaters.ID)

ParticipantNoSample.Centre <- Intake.DF %>% select(`Participant ID`,Centre) %>%
                               filter(`Participant ID` %in% ParticipantNoSample.ID)

ParticipantNoSample.PrimaryOutcome <- Outcome.DF %>% 
  select(`Participant ID`,PTB_Type_Formatted) %>%
  filter(`Participant ID` %in% ParticipantNoSample.ID)  %>%
  distinct()

Tmp <- merge(ParticipantNoSample.Centre,ParticipantNoSample.PrimaryOutcome,by="Participant ID",
             all = TRUE)

#Check if any double outcomes (there are none) 
length(ParticipantNoSample.ID) == nrow(Tmp)

ParticipantNoSample.Details <- Tmp %>% count(Centre,PTB_Type_Formatted) %>% 
                               dcast(PTB_Type_Formatted~Centre,value.var = "n",
                                     fill=0) %>%
                              rename(`Pregnancy Outcome` = PTB_Type_Formatted)

ParticipantNoSample.Details <- ParticipantNoSample.Details %>% 
  flextable() %>%
  flextable::line_spacing(space = 1.1, part = "body") %>% 
  fontsize(size = 9, part = "all") %>%
  padding(padding = 2, part = "all") %>%
  line_spacing(space = 0.9, part = "all") %>%
  width(j = 1, width = 2.0) %>%
  width(j = 2, width = 4.0) %>%
  set_table_properties(
    layout = "fixed",
    align = "center"
  )

```

### [**Figure 4**]{style="color: red;"}: Specimen Collection

```{r}
#| fig-width: 13
#| fig-height: 8

SampleCounts.Plot

```
::: {.callout-note appearance="note" title="**Figure 4**"}
Distribution of participants contributing each specimen type, stratified by primary pregnancy outcome as defined in the Pregnancy and Delivery Outcomes section. Within each outcome stratum, each coloured segment represents the number of distinct participants who contributed at least one specimen of the specified type during the relevant gestational sampling window. A participant who contributed multiple specimens of the same type within a given window is counted only once in that segment. However, participants may be counted in multiple segments if they contributed specimens during more than one gestational window. Consequently, the total length of a stacked bar represents the sum of the window-specific participant counts and does not necessarily equal the number of unique participants who contributed that specimen type. Facet scales are allowed to vary for readability. One participant who experienced miscarriage and contributed three specimens at their first and only visit was omitted from the plot for readability; complete sample collection counts, including data from this participant, are provided in Supplementary Table G.
:::

### [**Supplementary Table G**]{style="color: orange;"}: Sample Collection Counts

```{r}
DT::datatable(
  Sample_Details.df, extensions = "Buttons", rownames = FALSE,
  options = list(
    dom = "Bfrtip",
    buttons = list(list(extend = "copy",text = "Copy table")),
    pageLength = 10))

```

::: {.callout-note appearance="note" title="**Supplementary Table G**"}
Counts of participants contributing each specimen type, stratified by pregnancy outcome and gestational sampling window.
:::

### [**Supplementary Table H**]{style="color: orange;"}: Aliquot Collection Statistics

```{r}
DT::datatable(
  Aliquot_Details.df, extensions = "Buttons", rownames = FALSE,
  options = list(
    dom = "Bfrtip",
    buttons = list(list(extend = "copy",text = "Copy table")),
    pageLength = 10))

```

::: {.callout-note appearance="note" title="**Supplementary Table H**"}
Mean and median number of aliquots obtained from an individual sample collection, summarised by specimen type and specimen group. For example, this reflects the typical number of aliquots generated when a given specimen type, such as plasma, was collected from a participant.
:::

### [**Figure 5**]{style="color: red;"}: Participant Visit Pattern (UpSet Plot)

```{r}
#| fig-width: 10
#| fig-height: 6
UpsetPlot
```

::: {.callout-note appearance="note" title="**Figure 5**"}
 UpSet plot showing patterns of biological specimen contribution across gestational sampling windows in the INSIGHT cohort. Each set represents a gestational window in which participants contributed at least one biological specimen. Set size indicates the total number of participants who contributed a specimen within each window, irrespective of attendance at other windows. Intersection size indicates the number of participants who contributed specimens in each specific combination of gestational windows. Solid black circles indicate included sampling windows, and connecting black lines indicate participants who contributed specimens across more than one window.
:::


### [**Supplementary Table I**]{style="color: orange;"}: Participant Visit Pattern (Table)

```{r}
DT::datatable(
  Visit_Details.df, extensions = "Buttons", rownames = FALSE,
  options = list(
    dom = "Bfrtip",
    buttons = list(list(extend = "copy",text = "Copy table")),
    pageLength = 10))

```

::: {.callout-note appearance="note" title="**Supplementary Table I**"}
Participant attendance patterns across gestational sampling windows. Each row represents a distinct combination of completed sampling windows, with counts indicating the number of participants who contributed at least one biological specimen in those windows. Data is visualised in Figure 5.   \
   \
High-vaginal cell pellets were retained only from February 2015 onward, and low-vaginal specimens were introduced in May 2016. Consequently, availability/missingness for these sample types is partly structural and associated with participants’ year of recruitment. 
:::

### [**Supplementary Table J**]{style="color: orange;"}: Participants Without Samples

```{r}
ParticipantNoSample.Details
```

::: {.callout-note appearance="note" title="**Supplementary Table J**: Participants Without Samples"}
Pregnancy outcomes and recruitment sites for participants who were enrolled in the study but did not contribute biological specimens.
:::

# Software and Versions
```{r}
lock <- jsonlite::read_json("../renv.lock")

versions <- data.frame(
  Software = c("R", names(lock$Packages)),
  Version  = c(
    lock$R$Version,
    vapply(lock$Packages, `[[`, character(1), "Version")
  )
)

DT::datatable(
  versions, extensions = "Buttons", rownames = FALSE,
  options = list(
    dom = "Bfrtip",
    buttons = list(list(extend = "copy",text = "Copy table")),
    pageLength = 10))

```
```
